# A novel ROR1-targeting antibody-PROTAC conjugate promotes BRD4 degradation for solid tumor treatment

**DOI:** 10.7150/thno.102531

**Published:** 2025-01-01

**Authors:** Lei Wang, Yong Ke, Qunye He, Pameila Paerhati, Weiliang Zhuang, Yali Yue, Junjun Liu, Jiawei Zhang, Lulu Huang, Qiang Yin, Huifang Zong, Jianwei Zhu, Baohong Zhang

**Affiliations:** 1 Engineering Research Center of Cell & Therapeutic Antibody, Ministry of Education, School of Pharmacy, Shanghai Jiao Tong University, Shanghai 200240, China.; 2 Jecho Institute, Co. Ltd, Shanghai 200240, China.

**Keywords:** Targeted protein degradation, Degrader-antibody conjugate, Ubiquitin-proteasome system, PROTAC, BRD4.

## Abstract

**Rationale:** Proteolysis Targeting Chimeras (PROTACs) are bifunctional compounds that have been extensively studied for their role in targeted protein degradation (TPD). The capacity to degrade validated or undruggable targets provides PROTACs with significant potency in cancer therapy. However, the clinical application of PROTACs is limited by their poor *in vivo* potency and unfavorable pharmacokinetic properties.

**Methods:** In this study, a novel degrader-antibody conjugate (DAC) was developed by conjugating the BRD4-degrading PROTAC with the ROR1 (receptor tyrosine kinase-like orphan receptor 1) antibody. The *in vitro* affinity, internalization efficacy, degradation, and cytotoxic activity of the ROR1 DAC were assessed. The pharmacokinetics, antitumor activity, and acute toxicity of ROR1 DAC were evaluated in mouse models. RNA sequencing (RNA-seq) and immunohistochemistry were performed to analyze the therapeutic efficacy mediated by the combination of ROR1 DAC and anti-mouse programmed cell death protein 1 (αmPD1) mAb.

**Results:** The ROR1 DAC exhibited strong degradation activity and cytotoxicity following antigen binding and internalization. Compared to unconjugated PROTAC, the ROR1 DAC demonstrated improved pharmacokinetics and potent antitumor efficacy in PC3 and MDA-MB-231 xenograft mouse models. Furthermore, enhanced antitumor activity and immune cell infiltration within solid tumors were observed when combined with αmPD-1 mAb in C57BL/6J mice. RNA sequencing revealed that the enhanced immune response associated with the combination treatment is related to tumor microenvironment modulation, including the upregulation of Th1-biased cytokines. Moreover, the ROR1 DAC exhibited a favorable safety profile in an acute toxicity study.

**Conclusions:** These results indicate that the degrader-antibody conjugate is a promising candidate for tumor-specific degradation and effective cancer therapy.

## Introduction

Targeted protein degradation represents a promising therapeutic approach for cancer, degrading oncoproteins via proteasomal, lysosomal, or autophagy pathways 1-3. PROteolysis Targeting Chimeras, a heterobifunctional molecule, are typically composed of an E3 ubiquitin ligase binder, a protein of interest (POI) binder, and a linker connecting both binders. PROTACs can mediate the interaction between the POI and E3 ubiquitin ligase, thereby facilitating the ubiquitination and subsequent degradation of the POI via the ubiquitin-proteasome system 4, 5. The ability of PROTAC to degrade “undruggable” targets, coupled with its catalytic and event-driven pharmacology, provides distinct advantages over traditional small-molecule inhibitors 6. The degraders ARV-471 and ARV-110 have recently been developed and are currently in phase II clinical trials 7, 8. However, enhancing the pharmacokinetics and tumor specificity of PROTACs, which are currently suboptimal, remains a significant challenge.

As a member of the bromodomain and extra-terminal (BET) family, bromodomain-containing protein 4 (BRD4) interacts with hyper-acetylated histone lysine residues and participates in transcriptional and epigenetic regulation 9, 10. BRD4 inhibitor and degrader are regarded as promising epigenetic cancer therapies. Many clinical studies have been terminated due to adverse effects arising from the poor selectivity of pan-BRD4 inhibitors. Various strategies have been developed to achieve tumor-specific delivery and enhance antitumor potency, including antibody-PROTACs. 11, 12, aptamer-PROTAC conjugate 13, and POLY-PROTAC nanoparticles 14. Antibody-drug conjugate (ADC) comprises a monoclonal antibody, a cytotoxic payload, and a chemical linker, potentially reducing off-target toxicities and improving targeting specificity and pharmacokinetic properties 15-17. The conjugation of PROTAC with an antibody, commonly referred to as "degrader-antibody conjugate", represents a promising strategy to enhance on-target degradation activity 17-20. The field of degrader-antibody conjugates is advancing rapidly, with several DACs for cancer treatment having been validated *in vitro*. However, in-depth evaluations of their *in vivo* efficacy and efforts to explore other combination therapies with antitumor activity have not yet been conducted. Therefore, developing a novel degrader-antibody conjugate to overcome the undesirable pharmacokinetic (PK) properties of PROTAC and enhance its potential *in vivo* therapeutic efficacy is imperative.

As a cancer-associated antigen, ROR1 is highly expressed in certain hematological and solid tumors but is expressed at low levels in normal adult tissues 21, 22. Blocking the Wnt5A/ROR1 pathway using ROR1 mAb can induce apoptosis in cancer cells 23, 24, while naked mAb alone exhibit limited clinical antitumor efficacy 25. The expression profile and rapid internalization make ROR1 an excellent target for ADC development, which has entered the clinical stage 26. In this study, we generated a novel anti-ROR1 degrader antibody conjugate that selectively and efficiently degrades BRD4. Subsequently, we evaluated antigen binding, cell apoptosis, cytotoxicity, and internalization activities. The ROR1 DAC exhibited potent *in vivo* antitumor activity in PC3 and MDA-MB-231 xenograft mouse models. Furthermore, the combination of ROR1 DAC and αm-PD1 mAb demonstrated significant T cell infiltration in the MC38 mouse model. Additionally, an improved PK profile and reduced toxicity were observed due to the conjugation.

## Methods

### Cell lines and animals

HEK293T, Jeko-1, MDA-MB-231, MC38, MCF-7, and PC3 cells were obtained from the Cell Bank of the Chinese Academy of Sciences (Shanghai, China). The cells were cultured under standard conditions using the recommended medium provided by the supplier. Expi293F cells were obtained from Invitrogen (Carlsbad, CA, USA) and cultured in 293 Hi-exp medium (OPM Biosciences, Shanghai, China).

Female BALB/c nude mice, C57BL/6J mice, and BALB/c mice from Charles River company (Zhejiang, China) were fed under specific pathogen-free conditions. Animal experiments were conducted in accordance with the ethical guidelines of Shanghai Jiao Tong University.

### Generation of antibodies and conjugates

The variable region sequences of the anti-ROR1 light and heavy chains were obtained from patent US10900973B2. L234A, L235A, and P329G mutations were introduced into the constant region. The sequences of anti-SARS-COV2 isotype control antibody 5B2 were provided in previous studies 27. All genes were synthesized and cloned into the pcDNA3.4 expression vector from Generabiol (Chuzhou, China). Cysteine residue sites were introduced via overlap PCR. The plasmids were prepared and transfected into Expi293F cells for antibody transient gene expression as described in a previous study 28. The antibodies were purified using Mabselect Sure LX (Cytiva, Uppsala, Sweden) and dialyzed into PBS (pH 7.2).

The conjugates were generated using the antibodies with engineered reactive cysteine residues (THIOMABs) method, along with click chemistry 29. Briefly, thio-ROR1 mAb (5 mg/mL, 1 mL) was reduced by 30 molars excess tris (2-carboxyethyl) phosphine hydrochloride (TECP.HCl) (Merck, Billerica, MA, USA) for 2 h at 37 ℃. The reduced antibody was dialyzed in PBS (pH 7.2) to remove TECP.HCl, and then was re-oxidized using 15-20 molars of excess dehydroascorbic acid (DHAA, Merck, Billerica, MA, USA ) for 4 to 6 h. The antibody was purified using HiTrap SP FF cationic exchange column (Cytiva, Uppsala, Sweden) to remove DHAA and aggregates. After ultrafiltrating into PBS (pH 7.0), the antibody reacted with 10 molars excess Mal-PEG_4_-DBCO (Merck, Billerica, MA, USA), Mal-PEG_0_-DBCO (Qiyue Biology, Xi'an, China), Mal-PEG_8_-DBCO (Qiyue Biology, Xi'an, China) and EDTA (2 mM) at 4 ℃ overnight. Subsequently, the sample was dialyzed into PBS (pH 7.2) and reacted with 20 molars excess the azido-MZ1 (Energy Chemical, Shanghai, China) dissolved in DMF at 20 ℃ for 12-24 h, followed by additional DMF at a final concentration of 10-15%. The excess reagents were removed using G-25 desalting column (Cytiva, Uppsala, Sweden), and the conjugate was purified and subjected to ultra-filtrated into 20 mM histidine acetate, pH 5.5 with an addition of 0.1% Tween-80 and 9% trehalose.

### Western blotting analysis

After drug treatment in a 12-well plate, the wells were aspirated and washed with PBS. Cells were lysed with 100 μL cold Western and IP lysis buffer containing a 2% inhibitor cocktail (Beyotime, Shanghai, China) for 10 min on ice. After centrifugation (12,000 *g*, 10 min, 4 ℃), the supernatants were collected and quantified with bicinchoninic acid assay (BCA) assay kit (Beyotime, Shanghai, China). 20-30 μg total protein was separated by sodium dodecyl sulfate-polyacrylamide gel electrophoresis (SDS-PAGE) and transferred onto the polyvinylidene fluoride (PVDF) membrane. The membrane was subsequently washed with PBST (0.1% Tween-20) and incubated with 5% skim milk at room temperature for 2 h, BRD4 (E2A7X) rabbit mAb (Cell Signaling Technology, Danvers, MA, USA) or beta-actin antibody (Affinity, Liyang, China) at 4 ℃ overnight, anti-rabbit IgG-HRP-linked antibody (Cell Signaling Technology, Danvers, MA, USA) at room temperature for 1 h. Finally, the bands were detected using ECL reagent (NCM biotech, Suzhou, China) by Tanon-4600 imaging system (Shanghai, China).

### Construction of HiBiT-BRD4 system on PC3 cell

A lentiviral system carrying HiBiT-tagged BRD4 (GeneID: 23476) was employed to create a cell pool expressing HiBiT-BRD4. Briefly, HEK-293T cells were transfected with 6 μg of a cytomegalovirus promoter-driven pHIV-puro plasmid containing HiBiT-tagged BRD4 gene, 6 μg of the psPAX2 plasmid, and 2 μg of the VSV-G plasmid using Lipo8000 (Beyotime, Shanghai, China). 12 h later, the fresh medium was replaced in the dish. 48 h later, the lentiviral medium was collected, filtered with 0.45 μm sterile syringe filters, and concentrated with lentivirus concentration kit (Genomeditech, Shanghai, China). The PC3 cells in a 6-well plate were then infected with concentrated viral particles containing 10 μg/mL polybrene (Merck, Billerica, MA, USA) and selected using 2 μg/mL puromycin (Beyotime, Shanghai, China). Following drug treatment, the degradation level of HiBiT-BRD4 was measured using HiBiT lytic detection reagents (Promega, Madison, WI, USA).

### Enzyme-linked immunosorbent assay (ELISA) for determining antigen binding avidity

100 μL ROR1 antigen (1 μg/mL, AcroBio-systems, Beijing, China) were coated at 4 °C overnight. Each well was washed four times with PBST and subsequently incubated with 200 μL of 3% BSA at 37 °C for 2 h, followed by 100 μL of three-fold serially diluted antibodies at 37 °C for 1 h, 100 μL of goat anti-human IgG (Fc specific) peroxidase antibody (1:10,000 dilution, Merck, Billerica, MA, USA) at 37 °C for 1 h, and 100 μL of peroxidase substrate (Solarbio, Beijing, China) for 10 min in the dark, followed by 50 μL of stop buffer (Solarbio, Beijing, China). The absorbance at 450 nm was measured using a TECAN Infinite M200 Pro (Männedorf, Switzerland).

### Flow cytometry analysis

For antigen binding detection, 2 × 10^5^ cells per test were collected and incubated with serially diluted ROR1 mAb and ROR1 DAC for 30 min at 4 °C. The cells were washed three times with PBS containing 2% fetal bovine serum (FBS) and stained with phycoerythrin (PE) anti-human Fc antibody (BioLegend, San Diego, CA, USA) for 30 min at 4 °C. After washing, the mean fluorescence intensity (MFI) was measured using CytoFLEX S flow cytometer (Beckman Coulter, Miami, FL, USA).

For the ROR1 internalization assay, 2 × 10^5^ cells per test were collected and incubated with ROR1 mAb or ROR1 DAC at a final concentration of 1 μg/mL concentration. After 1 h incubation at 4 °C, cells were placed at 37 °C for 0.5 h, 1 h, 2 h, 4 h, and fixed with 2% paraformaldehyde for 30 min at 20 °C. The cells were incubated with PE anti-human Fc antibody for 30 min and detected by flow cytometry.

For apoptosis and cell cycle assays, each well in a 6-well plate was seeded with 4 × 10^5^ cells overnight. Cells were incubated with PBS, ROR1 mAb (100 nM), ROR1 DAC (100 nM), and MZ1 (1 μM) for four days, respectively. After collection, the cells were stained and analyzed using the Annexin V/ Propidium iodide (PI) apoptosis kit (Multi Sciences, Hangzhou, China) or cell cycle kit (Multi Sciences, Hangzhou, China).

### Internalization imaging assay

Antibodies were labeled using pHrodo^TM^ iFL Red STP labeling reagents (Thermo Fisher, Waltham, MA, USA). 1 × 10^5^ PC3 cells were plated in a 24-well plate overnight. Cells were treated separately with PBS, labeled ROR1 mAb (final concentration 150 nM), and pHrodo Red labeled ROR1 DAC (final concentration 150 nM) at 37 °C for 4 h, and stained with 1000 × LysoSensor Green DND-189 (Thermo Fisher, Waltham, MA, USA) for 40 min, 100 × Hoechst (Beyotime, Shanghai, China) at 37 °C for 10 min. Cells were washed with pre-warmed PBS and then were sent to acquired images by the Operetta CLS high content analysis system (PerkinElmer, Waltham, MA, USA). The colocalization overlap coefficients were analyzed using the ImageJ_JACoP software.

### Cell cytotoxicity assay

Five thousand tumor cells were seeded in 96-well plates and cultured overnight. ROR1 mAb, ROR1 DAC, and MZ1 were serially diluted and added to the plates. After four days of incubation, cell viability was assessed using a Cell Counting Kit-8 (CCK-8, Dojindo, Kumamoto, Japan).

### Pharmacokinetic assay

Five female BALB/c mice (8 weeks old) per group were treated intravenously with 10 mg/kg ROR1 DAC or ROR1 mAb, respectively. Serum samples were collected on days 0.25, 1, 2, 4, 7, 10, 15 and 21. The serum concentrations of ROR1 DAC and mAb were measured using ELISA. The goat anti-human IgG kappa chain-specific antibody (Merck, Billerica, MA, USA) was coated as the capture antibody, and the goat anti-human IgG (Fc specific)-peroxidase antibody (Merck, Billerica, MA, USA) was used as the detection antibody. The PK parameters of ROR1 mAb and ROR1 DAC were analyzed using a non-compartmental model with the PK Solver 2.0 software.

### *In vivo* antitumor efficacy study

The *in vivo* efficacy of ROR1 DAC was evaluated using three xenograft mouse models. In the PC3 and MDA-MB-231 xenograft models using BALB/c nude mice, thirty female mice were randomly divided into six groups (n = 5) based on body weight. 5 × 10^6^ cells in 100 μL of cold culture medium were subcutaneously implanted into the right flank of each mouse. When the tumor reached a volume of 50 - 100 mm^3^, mice were intravenously treated with PBS, ROR1 mAb (15 mg/kg), Isotype DAC control (15 mg/kg), ROR1 DAC (5 or 15 mg/kg), and MZ1 (5 mg/kg) every five days. The body weight and tumor volume were measured twice a week. The tumor volume was calculated by the formula: volume (mm^3^) = length × width^2^/2. Before dissection, serum samples were collected for biochemical index analysis using the Mindray Chemistry Analyzer BS360S, and the tumors were excised, weighed, and photographed.

In the MC38 xenograft model, thirty-five C57BL/6J female mice were divided into seven groups (n = 5) based on body weight. MC38 cells stably expressing the human ROR1 antigen were generated using a lentiviral system and cultured following single-cell isolation via FACSAria II (BD, San Jose, CA, USA). 2 × 10^5^ cells in 100 μL cold of culture medium were subcutaneously implanted into the right flank of each mouse. When the tumors reached a volume of 50-100 mm³, the mice were treated intravenously with PBS, ROR1 mAb (15 mg/kg), Isotype DAC control (15 mg/kg), ROR1 DAC (15 mg/kg), MZ1 (5 mg/kg), anti-mouse PD-1 mAb (αmPD-1 mAb, RMP1-14, BioXCell, Lebanon, NH, USA) (5 mg/kg), and αmPD-1 mAb (5 mg/kg) in combination with ROR1 DAC (15 mg/kg) every three days. The tumor volume and body weight were measured twice a week. One day before the end of the experiment, blood samples were collected for biochemical index analysis. The tumors were stripped and weighted under nuclease-free conditions. Then, the stripped tumors were cut and divided for western blotting, RNA-seq and immunohistochemistry analysis. The mouse BRD4 was detected using BRD4 polyclonal antibody (Proteintech, Wuhan, China). The lymphocytes were separated from the spleen using the mouse lymphocyte separation medium (Dakewe, Shenzhen, China). Cells were stained with PE rat anti-mouse CD4, Fluorescein Isothiocyanate (FITC) rat anti-mouse CD8a, and Allophycocyanin (APC) hamster anti-mouse CD3e (BD, San Diego, CA, USA) and analyzed by flow cytometry.

### RNA sequencing

Total mRNA was extracted from the MC38-rhROR1 tumor. The purity and concentration of the extracted mRNA were measured using a NanoDrop 2000 spectrophotometer. cDNA library construction, sequencing using NovaSeq X Plus, and alignment to the mouse (GRCm39) reference genome using Bowtie2 were conducted by Shanghai Majorbio Bio-pharm Technology Co., Ltd. Differentially expressed genes (DEGs) were identified based on the criteria of a P-value of 0.05 and a log2 (fold change, FC) > 2. Gene ontology (GO), Kyoto Encyclopedia of Genes and Genomes (KEGG), Reactome enrichment as well as hierarchical clustering analysis, were performed using the online tool of Majorbio Cloud Platform. Gene Set Enrichment Analysis (GSEA) was performed using GSEA software version 4.3.2. The abundance of immune cells was analyzed using Immune Cell Abundance Identifier for mouse (ImmuCellAI_mouse, https://guolab.wchscu.cn/ImmuCellAI-mouse). Real-time quantitative PCR was performed using Hieff qPCR SYBR Green Master Mix (Takara, Dalian, China) on CFX Opus 96 Real-Time PCR System (Bio-Rad, Hercules, CA, USA). The primer sequences are listed in Table S1.

### Acute toxicity analysis of ROR1 DAC

Female BALB/c mice (8 weeks old) were divided into five groups (n = 5) and administered single doses of 100 mg/kg ROR1 DAC, 50 mg/kg ROR1 DAC, PBS, and two doses of 30 mg/kg MZ1, along with a vehicle control based on the PK parameters. Body weight was measured daily. Blood samples were conducted on days 1, 4, and 7 for the blood routine examination and liver and kidney function assessment. Hematological indicators, including platelets (PLT), red blood cells (RBC), and white blood cells (WBC), were examined using the Sysmex Hemostasis Analyzer XN-1000V (B1). Indicators related to liver and renal functions, including aspartate aminotransferase (AST), alanine aminotransferase (ALT), and urea (UREA), were detected using the Mindray Chemistry Analyzer BS360S. On day 8, mice were euthanized via carbon dioxide inhalation. The liver, spleen, kidneys, lungs, and heart were excised for damage analysis using hematoxylin and eosin staining (H&E staining).

### Statistical analysis

Data were analyzed using GraphPad Prism Software 8.3.0. Statistically significant differences were assessed using one-way ANOVA and unpaired t-tests. Group data are presented as mean ± SEM. A p-value of < 0.05 was considered statistically significant.

## Results

### Optimizing conjugation sites and linkers for the generation of ROR1-BRD4 degrader conjugates

**Figure 1:**
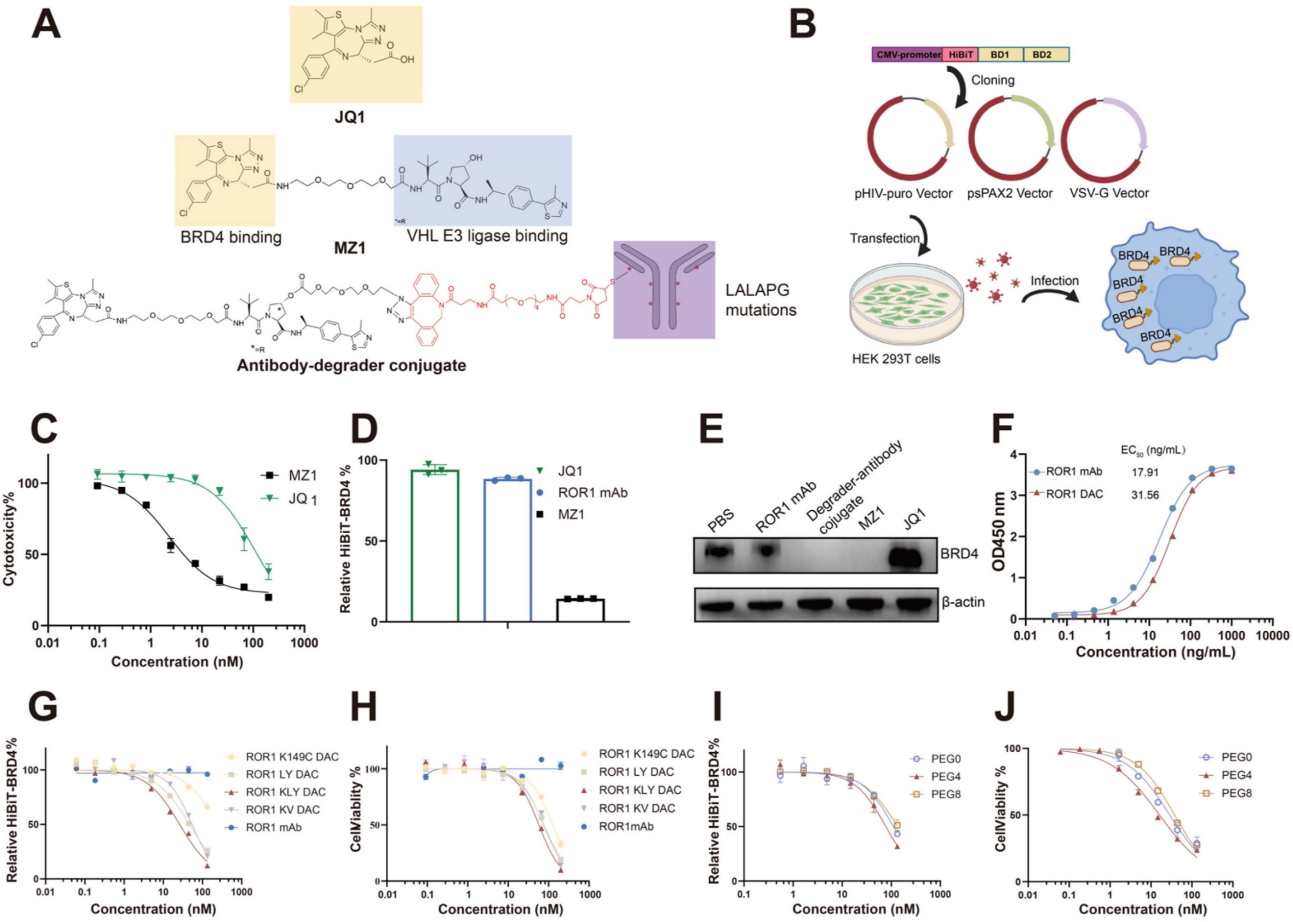
** Generation and optimization of ROR1-BRD4 degrader conjugate. (A)** Structures of compound MZ1 and degrader-antibody conjugate. The ADCC and CDC functions of ROR1 mAbs were reduced with mutations of L234A, L235A, and P329G on the Fc region. The hydroxyl group of VHL is modified with an azido group and tethered to a maleimide-PEG-DBCO linker, which is then conjugated to ROR1 mAb with mutated cysteine. **(B)** Cartoon illustration of the construction of PC3 cell pool stable expressing HiBiT-BRD4. The HiBiT-tag was fused to the N-terminal of the BRD4 coding sequence and subsequently cloned into the pHIV vector. Lentiviruses generated using second-generation lentiviral vectors were used to infect PC3 cells, followed by selecting stable cell pools under puromycin dihydrochloride pressure. An illustration was created with BioRender.com. **(C)** The cytotoxicity assay of MZ1 and JQ1 using CCK-8 kit. Cells were treated with serial-diluted JQ1 and MZ1 for four days. Cell viability was detected through the CCK-8 kit.** (D)** Relative BRD4-HiBiT level analysis of different groups. 100 nM ROR1 mAb, 1 μM JQ1, and 1 μM MZ1 were added to the PC3 cells expressing HiBiT-BRD4 for 6 h. The supernatants were removed, followed by a HiBiT-BRD4 level detection using the HiBiT lytic detection system.** (E)** Western blotting analysis of BRD4 and β-actin in cells. Cells were incubated with the 100 nM ROR1 mAb, 100 nM ROR1 DAC, 1 μM MZ1, and 1 μM JQ1 for 4 h before harvesting cell lysates. **(F)** ROR1 antigen-binding ELISA assay of ROR1 mAb and ROR1 conjugate with different concentrations. HiBiT-BRD4 degradation efficacy assay of DAC with different conjugation sites **(G)** and lengths of PEG linker **(I).** Serially diluted DACs were added to the PC3 cells expressing HiBiT-BRD4 for 8 h. The supernatants were removed, followed by a HiBiT-BRD4 level detection using the HiBiT lytic detection system. The cytotoxicity assay of DACs with different conjugation sites** (H)** and lengths of PEG linker **(J)**. Serially diluted DACs were added to the PC3 cells for four days, followed by detection of cell viability using CCK-8. EC_50_ values were calculated by GraphPad Prism software 8.3.0. Mean was shown as SEM.

MZ1 consists of the BRD4 binding ligand JQ1 and the VHL ligase binding ligand VHL032 (Figure 1A). We compared the cytotoxicity of JQ1 and MZ1 using the CCK-8 assay in PC3 cells. The CCK-8 assay indicated that the IC_50_ value for JQ1 was 112.3 nM, approximately 49 times higher than that of MZ1, which had an IC_50_ value of 2.292 nM. HiBiT, an 11-amino acid peptide fused to BRD4, exhibits luminescence and bright NanoBiT luciferase upon complementation with LgBiT 33. The BRD4 protein fused to HiBiT at its N-terminal was expressed in PC3 cells using a lentiviral system, allowing for precise and quantitative detection of BRD4 degradation (Figure 1B). The low cytotoxicity and apoptosis activity of JQ1, attributed to its lack of BRD4 degradation activity, renders it an unsuitable payload for the development of antibody-drug conjugates (Figure 1C, Figure S1). The HiBiT assay results indicated that JQ1 could not degrade BRD4, whereas MZ1 was effective under the same experimental conditions (Figure 1D). The western blotting also verified that JQ1 could not degrade the BRD4 after 4 h incubation on PC3 cells, while the MZ1 and degrader-antibody conjugate could significantly degrade the BRD4 (Figure 1E). The degrader-antibody conjugate consists of antibody, linker, and degrader. For DAC generation, antigen selection should meet the criteria for tumor-associated expression and internalization while being consistent with the expression profile of the POI 30. The DAC circulating in the body specifically binds to the antigen and is subsequently transported into the targeted cell. The degraders are released in the lysosome and transported out, allowing them to degrade the POI through the UPS. The uniform expression of ROR1 in cancer, along with its internalization capability and strong correlation with BRD4, makes it a promising target for conjugation. We assessed the correlation coefficients between the levels of BRD4 and ROR1 using data from The Cancer Genome Atlas. Our findings revealed a statistically significant positive correlation between BRD4 and ROR1, particularly in prostate adenocarcinoma and invasive breast carcinoma (Figure S2). The hydroxyl group of MZ1 was linked to the azido group for the click reaction and maleimide for subsequent site-specific conjugation. The ROR1 antigen-binding ELISA results showed the binding activity of ROR1 conjugates was slightly lower than the parental antibody (Figure 1F).

The conjugation sites on antibodies can affect the drug homogeneity, stability, and therapeutic efficacy 31, 32. The optimization of various DACs was evaluated using the HiBiT degradation detection system and CCK8 assay. We employed maleimide-PEG_4_-DBCO as a linker to compare the degradation efficacy and cytotoxicity of different mutated cysteine sites. The conjugate with the triple mutations LC-K149C, HC-L174C, and HC-Y373C demonstrated the lowest DC_50_, which was nine-fold lower than the single mutation and 1.7-fold lower than the dual mutations. Additionally, it also exhibited the lowest IC_50_ value (Figure 1G, H, Table S2), consistent with the former report 11. Additionally, we evaluated whether the different PEG lengths of the linker could influence degradation efficacy and cytotoxicity. The linker with four PEG units exhibited better degradation efficacy and cytotoxicity (Figure 1I, J). Therefore, ROR1 mAb with LC-K149C, HC-L174C, and HC-Y373C mutation sites, utilizing maleimide-PEG_4_-DBCO as linker was investigated in subsequent experiments, referred to as “ROR1 DAC” in this study.

### ROR1 DAC degrades BRD4 in a UPS-dependent manner

**Figure 2:**
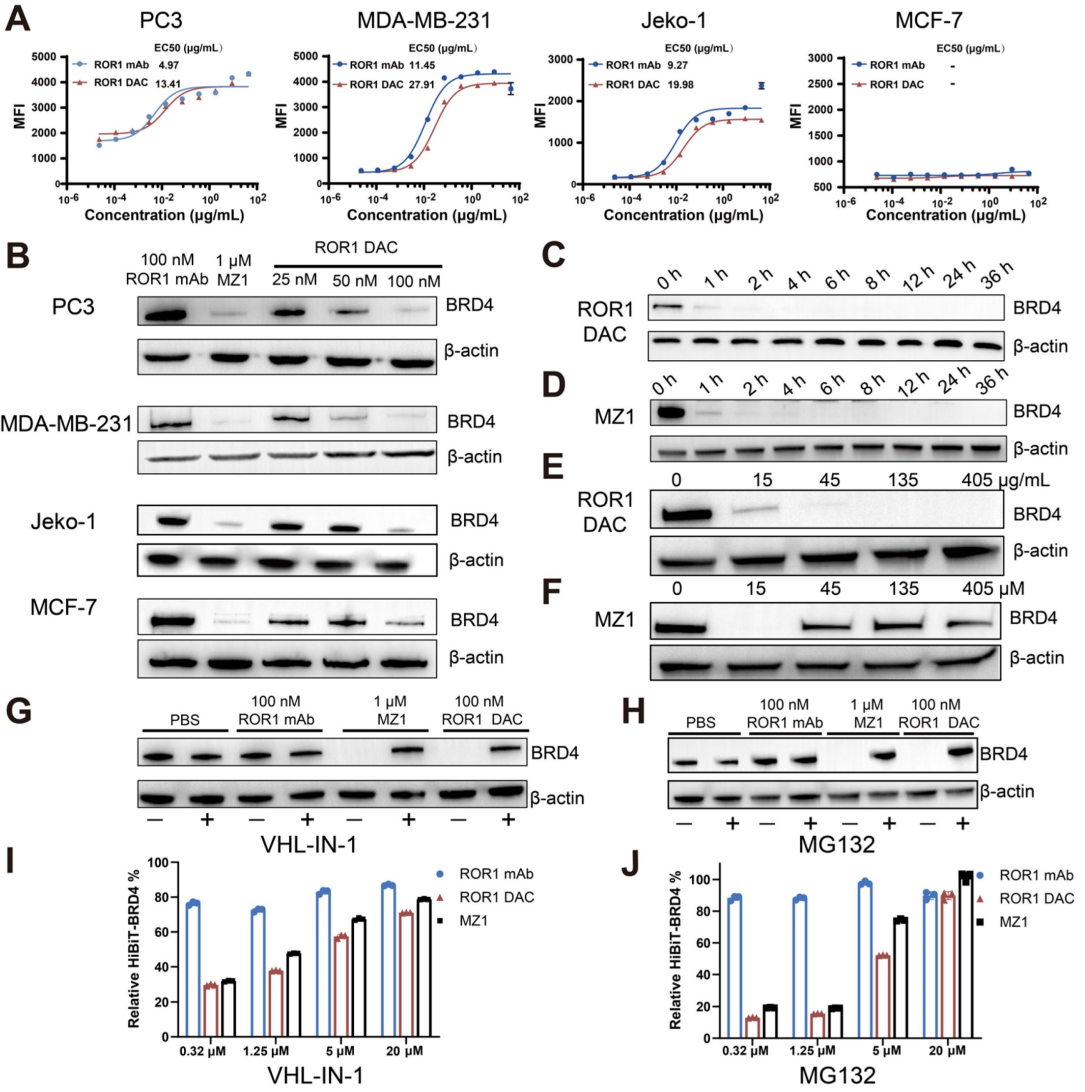
** The antigen-specific BRD4 degradation mediated by ROR1 DAC depends on the UPS system. (A)** Cell binding curve of ROR1 DAC and ROR1 mAb to PC3, MDA-MB-231, Jeko-1, and MCF-7 cells. The median fluorescence intensities (MFI) were detected by flow cytometry, and EC_50_ was calculated using GraphPad Prism 8.3.0. Data are presented as mean ± SEM with three replicates.** (B)** Western blotting analysis of BRD4 and β-actin in PC3, MDA-MB-231, Jeko-1, MCF-7 cells. Cells were incubated with the indicated concentrations of ROR1 mAb, MZ1, and ROR1 DAC for 4 h before harvesting cell lysates. Analysis of BRD4 degradation mediated by ROR1 DAC** (C)** or MZ1**(D)** at different time points. PC3 cells were treated with 100 nM ROR1 DAC or 1 μM MZ1 at different time intervals before harvesting cell lysates. The lysates were subjected to BRD4 and β-actin analysis by western blotting. Hook effect analysis of ROR1 DAC **(E)** or MZ1**(F)**. PC3 cells were incubated with indicated concentrations of ROR1 DAC or MZ1 for 4 h before harvesting cell lysates. Western blotting analysis of BRD4 degradation mediated by ROR1 DAC is reversed by VHL inhibitor **(G)** and proteasome inhibitor **(H)**: PC3 cells were incubated with PBS, 100 nM ROR1 mAb, 1μM MZ1, and 100 nM ROR1 DAC for 4 h with (+) or without (-) 20 μM VHL-IN-1 or 20 μM MG132. HiBiT assay of BRD4 degradation mediated by ROR1 DAC is reversed by VHL inhibitor **(I)** and proteasome inhibitor **(J)**: PC3 cells were incubated with PBS, 100 nM ROR1 mAb, 100 nM ROR1 DAC, and 1μM MZ1 for 6 h with (+) or without (-) indicated concentration of VHL-IN-1 inhibitor or MG132 inhibitor.

Several cell lines exhibiting varying levels of ROR1 expression were selected for the ROR1 DAC binding activity assay. Flow cytometry results indicated that the MDA-MB-231 (triple-negative breast cancer, TNBC), Jeko-1 (mantle cell lymphoma), and PC3 (prostate cancer) cell lines were ROR1-positive. Additionally, the MCF-7 breast cancer cell line was identified as ROR1-negative (Figure S3). The results demonstrated that ROR1 DAC exhibited similar fluorescence intensities and binding curves to the parental ROR1 mAb in PC3, MDA-MB-231, and Jeko-1 cells, but did not bind to MCF-7 cells, indicating that the conjugation did not affect selectivity (Figure 2A). Western blotting results confirmed that the ROR1 DAC could strongly degrade the BRD4 on PC3, MDA-MB-231, and Jeko-1 cells in a dose-dependent manner, however, it could not degrade the BRD4 on MCF-7 cells (Figure 2B). Nearly complete degradation of BRD4 mediated by ROR1 DAC and MZ1 occurred at 4 h and did not recover over 36 h (Figure 2C, D). These results indicate that ROR1 DAC can effectively degrade BRD4 and sustain this degradation over 36 h. We also assessed the hook effect of ROR1 DAC at 27-fold the working concentration. The results indicated that ROR1 DAC was capable of degrading BRD4 at concentrations ranging from 15 to 405 μg/mL, which implies that no hook effect was present in this concentration range (Figure 2E). Furthermore, MZ1 exhibited a hook effect at a concentration of 45 to 405 μM (Figure 2F).

MZ1 can bind to both BRD4 and VHL E3 ligase, inducing the ubiquitination of BRD4, which ultimately leads to its degradation by the endogenous 26S proteasome. To validate the degradation mediated by ROR1 DAC was dependent on VHL E3 ligase. The result showed the degradation of BRD4 was inhibited by the VHL-IN-1 (Figure 2G). Additionally, PC3 cells were treated with ROR1 DAC both in the presence and absence of the proteasome inhibitor MG132, indicating that BRD4 degradation induced by ROR1 DAC is dependent on the proteasome (Figure 2H). The HiBiT assay indicated that as the concentrations of VHL-IN-1 or MG132 increase, the relative BRD4-HiBiT signal also increases, suggesting that ROR1 DAC-mediated degradation was inhibited (Figure 2I, J). Quantification of western blot data is shown in Figure S4. Taken together, these results confirm that the degradation induced by ROR1 DAC is dependent on the ubiquitin-proteasome pathway.

### Internalization and *in vitro* cytotoxicity assessment of ROR1 DAC

**Figure 3:**
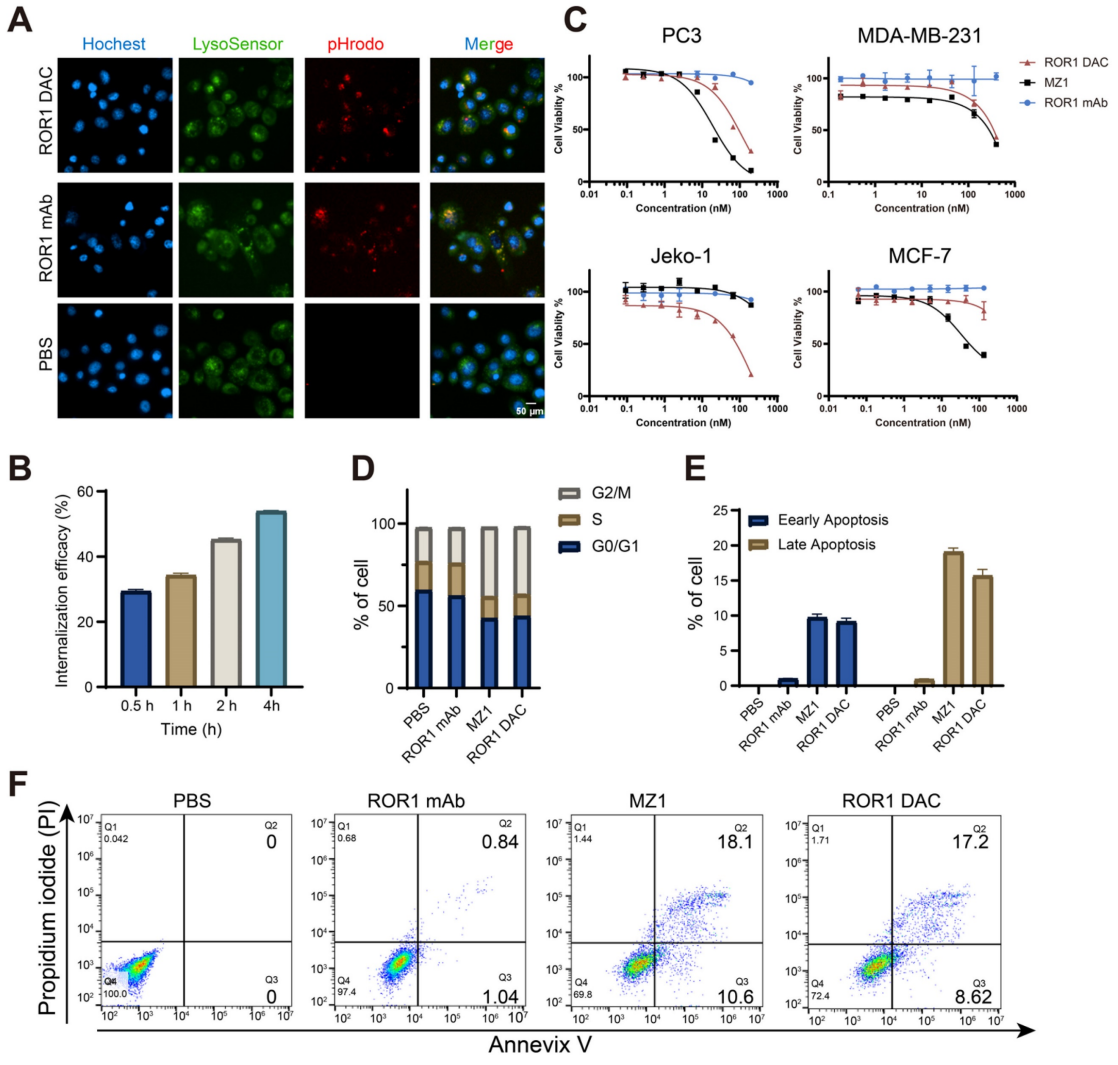
*** In vitro* internalization and cytotoxicity activity of ROR1 DAC. (A)** Internalization imaging examination of ROR1 DAC. PC3 cells were treated with pHrodo labeled PBS, 150 nM ROR1 mAb, and 150 nM ROR1 DAC for 4 h. Subsequent staining was performed with LysoSensor for 40 min and Hoechst solution for 10 min. Scale bar: 50 µm. **(B)** Internalization efficiency assay of ROR1 DAC in PC3 cells. Cells were treated with 1 μg/mL ROR1 DAC for the indicated time, followed by fixation and stained with PE-labeled antibody. Flow cytometry detected the MFI values and internalization efficiency was calculated based on samples fixed at 0 h. **(C)** Cytotoxicity assay of ROR1 DAC on PC3, MDA-MB-231, Jelo-1, and MCF-7 cells. Cells were treated with serial-diluted ROR1 DAC and MZ1 for four days. Cell viability was detected through the CCK-8 kit. Mean was shown as SEM with duplicates. **(D)** Cell cycle assay of PC3 cells by flow cytometry. Cells were incubated with PBS, 100 nM ROR1 mAb, 1 μM MZ1, and 100 nM ROR1 DAC for three days. After flow cytometry detection, the cell cycle was analyzed by Flowjo.V10. **(E)** The histogram of early and late apoptotic cells. **(F)** Apoptosis assay of PC3 cells by flow cytometry. Cells were incubated with PBS, 100 nM ROR1 mAb, 1 μM MZ1, and 100 nM ROR1 DAC for four days. Cells were collected and subjected to analysis of Annexin V-PI. Data was analyzed by Flowjo.V10.

The target-specific internalization and cytotoxicity of ROR1 DAC were subsequently investigated. To verify the internalization properties of ROR1 DAC, antibodies were labeled using the pHrodo^TM^ iFL Red STP labeling reagents, and red fluorescence was observed exclusively under acidic conditions, such as lysosomes or endosomes. The images confirmed that ROR1 DAC was internalized via the lysosome (Figure 3A, Figure S5). The colocalization overlap coefficients for ROR1 mAb and ROR1 DAC were determined to be 73.8% and 82.8%. The reduction of cell surface ROR1 on PC3 cells was measured by flow cytometry following drug incubation. The results indicated that nearly half of the ROR1 antigen was internalized after 4 h (Figure 3B). Following a four-day incubation with ROR1 mAb, ROR1 DAC, and MZ1, cell viability was assessed using the CCK-8 kit. MZ1 exhibited potent cytotoxicity across all cell lines, whereas ROR1 DAC demonstrated comparable cytotoxicity in ROR1-positive cells and limited cytotoxic activity at high concentrations in MCF-7 cells (Figure 3C, Table S3). Flow cytometry measurements revealed a significantly elevated percentage of cells in the G2/M phase of the cell cycle (Figure 3D), as well as an increased rate of apoptotic cell death induced by ROR1 DAC compared to control groups (Figures 3E, F).

### ROR1 DAC exhibits improved *in vivo* antitumor activity in PC3 and MDA-MB-231 xenograft mouse model

**Figure 4:**
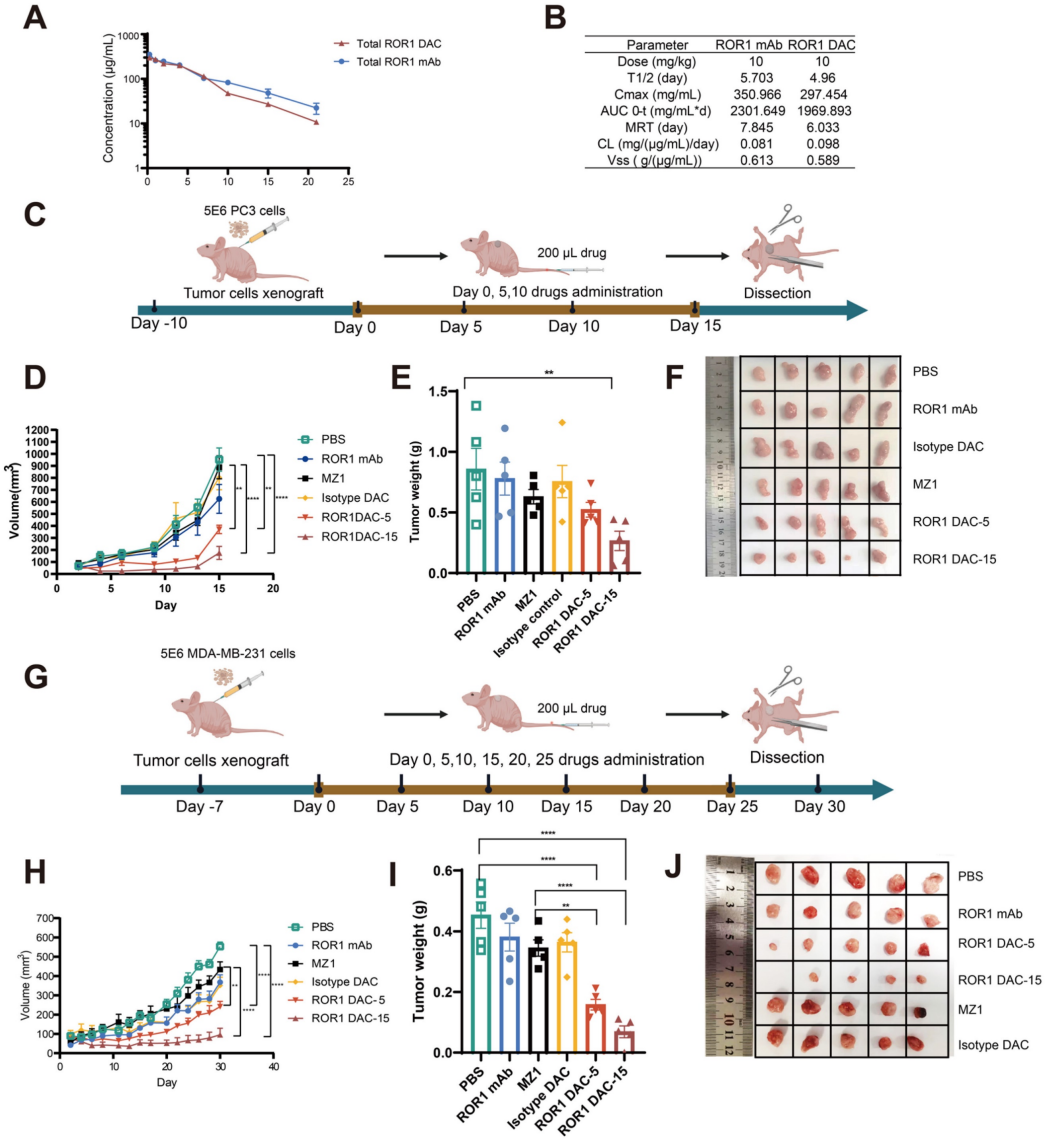
** Antitumor effect of ROR1 DAC in PC3 and MDA-MB-231 xenograft mouse models. (A)** The pharmacokinetics analysis of ROR1 mAb and ROR1 DAC in BALB/c mice. Mice (n = 5) were intravenously injected with 10 mg/kg of ROR1 mAb or ROR1 DAC, respectively. Serum samples were collected on the indicated days. ELISA was used to determine the concentrations of total DAC and mAb. **(B)** The pharmacokinetic parameters of ROR1 mAb and ROR1 DAC were calculated using PKSlover 2.0 software. **(C)** Schematic representation of tumor inoculation and drug treatment.** (D)** PC3 tumor volume changes across different groups. 5 × 10^6^ cells were implanted on the right flank of the mouse. PBS, ROR1 mAb (15 mg/kg), Isotype DAC (15 mg/kg), MZ1 (5 mg/kg), and ROR1 DAC (5 or 15 mg/kg) were administered when the tumor volume reached 50-100 mm^3^ every five days (n = 5). Histogram **(E)** and picture **(F)** of stripped tumor weight on PC3 xenograft mouse model. **(G)** Schematic representation of tumor inoculation and drug treatment on MDA-MB-231 xenograft mouse model. **(H)** MDA-MB-231 tumor volume changes across different groups. 5 × 10^6^ cells were implanted on the right flank of the mouse. PBS, ROR1 mAb (15 mg/kg), Isotype DAC (15 mg/kg), MZ1 (5 mg/kg), and ROR1 DAC (5 or 15 mg/kg) were administered when the tumor volume reached 50-100 mm^3^ every five days (n = 5). Histogram **(I)** and picture **(J)** of stripped tumor weight on the MDA-MB-231 model. Data were analyzed by One-way ANOVA and shown as Mean ± SEM. ns *P* > 0.05; * *P* < 0.05; **P < 0.01; ***P < 0.001; ****P < 0.0001.

The pharmacokinetic profiles of ROR1 DAC were evaluated to design the *in vivo* drug administration regimen rationally. The results indicated that ROR1 DAC exhibited a relatively long half-life of approximately 4.96 days (Figure 4A). Furthermore, the pharmacokinetic parameters of total ROR1 DAC were similar to those of the parental ROR1 mAb, suggesting a significant extension in the half-life of PROTAC that may enhance its therapeutic efficacy *in vivo* (Figure 4B). LC/MS analysis showed that the ROR1 DAC maintained a drug-to-antibody ratio of 4.98, 4.91, 4.76, 4.71, and 4.64 on days 0, 1, 3, 7, and 14, respectively, indicating relative stability in mouse serum over 7 days (Figure S6).

The antitumor effect of ROR1 DAC was investigated in the PC3 xenograft BALB/c nude mouse model. Mice (n = 5) were intravenously treated with different dosages of indicated drugs for four doses every five days when tumor volume reached approximately 100 mm^3^ (Figure 4C). Both dosages of ROR1 DAC significantly inhibited tumor growth compared to PBS, ROR1 mAb control, MZ1, and isotype DAC control (Figure 4D). Meanwhile, the average tumor weight in the two ROR1 DAC dosage groups was significantly lower than that of the other groups (Figure 4E, F). Notably, treatment with a comparable dose of unconjugated MZ1 showed no efficacy. However, the antitumor effect of the DAC groups diminished after drug discontinuation. ROR1 DAC did not induce significant weight changes in the two groups following drug administration (Figure S7A). The liver and renal function biochemical indices for all groups were within the standard range (Figure S7B). Additionally, the degradation of BRD4 mediated by ROR1 DAC was observed compared to the PBS and MZ1 groups (Figure S8).

We evaluated the ROR1 DAC in the MDA-MB-231 xenograft BALB/c nude mouse model to further investigate the antigen-dependent antitumor activity. Based on the *in vitro* cytotoxicity observed in MDA-MB-231 cells, we continuously administered six doses of the indicated drugs every five days when the tumor size reached approximately 100 mm^3^ (Figure 4G). Consistent with the results from the PC3 xenograft mouse model, two doses of ROR1 DAC significantly inhibited tumor growth compared to the control groups. However, the 5 mg/kg ROR1 DAC group could not effectively inhibit tumor growth compared to the 15 mg/kg ROR1 DAC group (Figure 4H-J). The isotype DAC group also demonstrated better *in vivo* activity than the MZ1 group, potentially due to the longer half-life mediated by the Fc domain of the antibody. ROR1 DAC did not induce significant changes in body weight or biochemical indices (Figure S9). These results demonstrate that ROR1 DAC conjugates significantly enhance MZ1's *in vivo* antitumor activity with high potency and are well tolerated in both xenograft models.

### The combination of ROR1 DAC with αmPD-1 mAb exerts synergistic antitumor effects in MC38 xenograft mouse model

The limited efficacy of immune checkpoint blockade by antibodies in various cancers may be partly attributed to a lack of immune cell infiltration and immune suppression within the tumor microenvironment. Recent studies have revealed that BET inhibitors can enhance antitumor immunity by suppressing programmed cell death ligand-1 (PD-L1) expression, a critical factor in cancer's immune evasion 33. The combination of BRD4 inhibitors with anti-PD1 mAb therapy suggests a hypothesis that this synergistic approach will not only target the drivers of tumor growth and survival but strengthen a robust and effective antitumor immune response.

**Figure 5:**
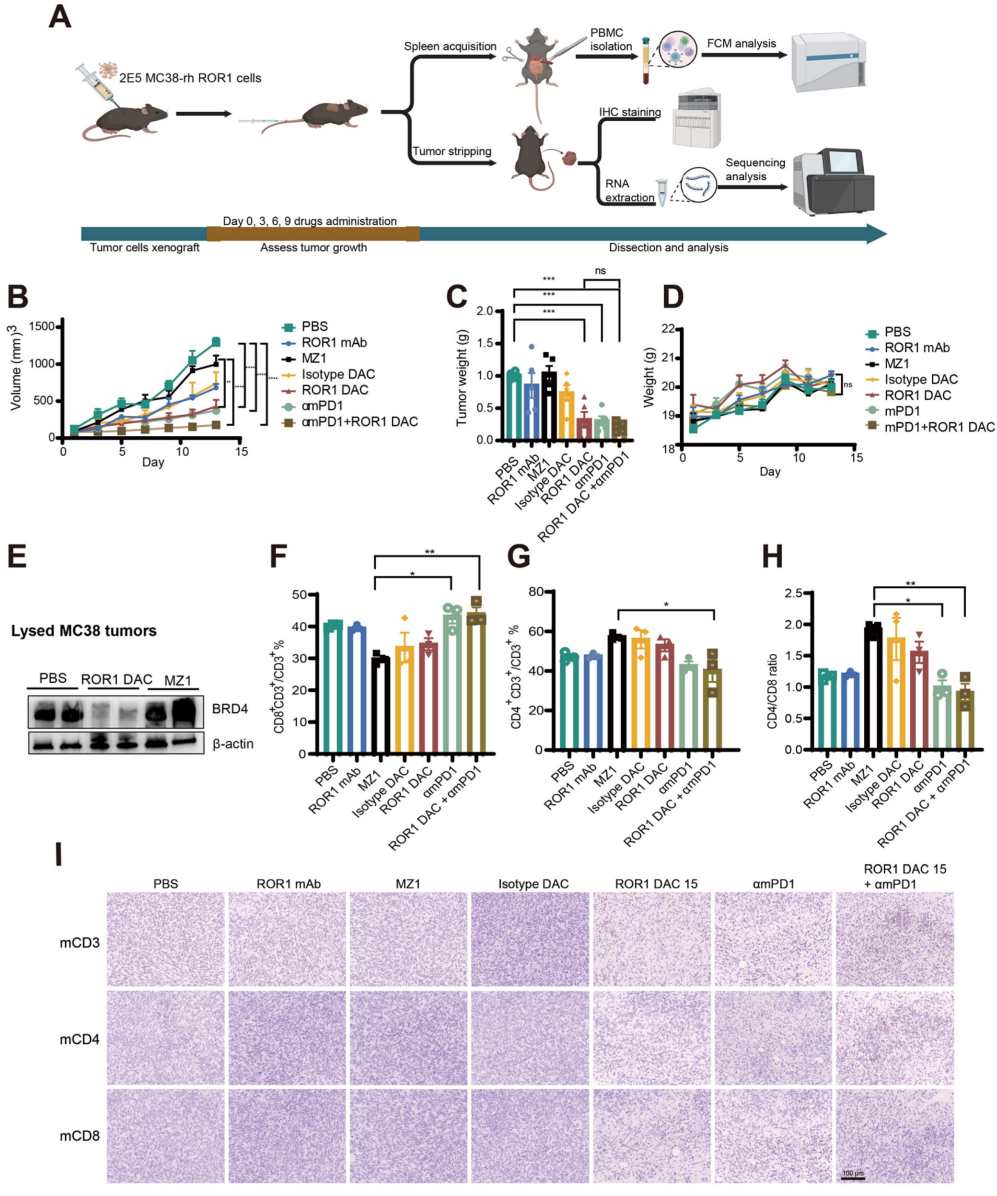
** Antitumor effect of ROR1 DAC combined with αmPD1 antibody on MC38-rhROR1 C57BL/6J mouse model. (A)** Schematic representation of tumor inoculation, drug treatment, and analysis. **(B)** Tumor volume changes across different groups. 2 × 10^5^ cells were subcutaneously implanted and monitored every two days. Drugs were administered via the tail vein when the tumor volume reached 50-100 mm^3^ every three days (n = 5). **(C)** Histogram of stripped tumor weight. **(D)** Changes in body-weight changes in different groups were monitored every two days. **(E)** BRD4 level detection of lysed MC38-rhROR1 tumors. Flow cytometry analysis of CD8+ percentage of CD3^+^ cells **(F)** and CD4^+^ percentage of CD3^+^ cells **(G)** after cell isolation from the spleen.** (H)** Ratio of CD4^+^/CD8^+^ T cells. **(I)** Immunohistochemistry analysis of mouse CD3, CD4, and CD8 in stripped tumors. Scale bar: 100 µm. Data were analyzed by One-way ANOVA and shown as Mean ± SEM. ns* P* > 0.05; * *P* < 0.05; **P < 0.01; ***P < 0.001; ****P < 0.0001.

The wild-type C57BL/6J mouse model, possessing an intact immune system, was selected to evaluate the efficacy and immune responses of ROR1 DAC. A stable MC38-rhROR1 monoclonal cell line, 2B10, expressing human ROR1 on the cell surface, was generated using a lentiviral system and sorted via flow cytometry (Figure S10). The 2B10 MC38-rhROR1 cells were implanted subcutaneously into C57/6J mice. At the endpoint of the experiment, immune responses were assessed using flow cytometry, immunohistochemistry staining, and RNA-seq (Figure 5A). ROR1 DAC, αmPD-1, and combination treatment exhibited a superior inhibitory effect compared to PBS, ROR1 mAb, and MZ1 groups (Figure 5B). The tumor weights in the combination treatment group were lower than those of other groups (Figure 5C). During the drug administration period, the increase in body weight of mice treated with ROR1 DAC was consistent with that of the control groups (Figure 5D), and the biochemical indices were within normal ranges (Figure S11), suggesting that combination therapy did not cause severe toxicity. At the end of the experiment, tumors were lysed for BRD4-level assessment via western blotting. The results revealed significant degradation of BRD4 in the ROR1 DAC group compared to both the PBS and MZ1 groups (Figure 5E). Quantification of western blot data is shown in Figure S4. PBMCs (Peripheral Blood Mononuclear Cells) from the spleen were collected and stained with mCD3, mCD4, and mCD8. Flow cytometry results indicated that αmPD-1 mAb therapy and combination therapy significantly enhanced the percentage of CD8^+^/CD3^+^ T cells and the CD4/CD8 ratio compared to MZ1, suggesting that the combination of PD-1 mAb may promote antitumor effects (Figure 5F-H). Additionally, immune cell infiltration in tumors was assessed using immunohistochemical staining for mCD3, mCD4, and mCD8. The results demonstrated a significant increase in tumor-infiltrating T cells in the ROR1 DAC, αmPD-1, and combination groups, with a notable increase in the combination group (Figure 5I). Only a few CD3-positive T cells were observed in the ROR1 mAb groups, with almost none detected in the PBS, isotype DAC, and MZ1 groups. Given that the C57BL/6J mouse model can objectively reflect immune responses, these results indicate that ROR1 DAC treatment can engage T cells to suppress tumor growth, while PD-1 blockade further enhances antitumor activity.

### The combination of ROR1 DAC and αmPD-1 mAb treatment enhances the immune responses

**Figure 6:**
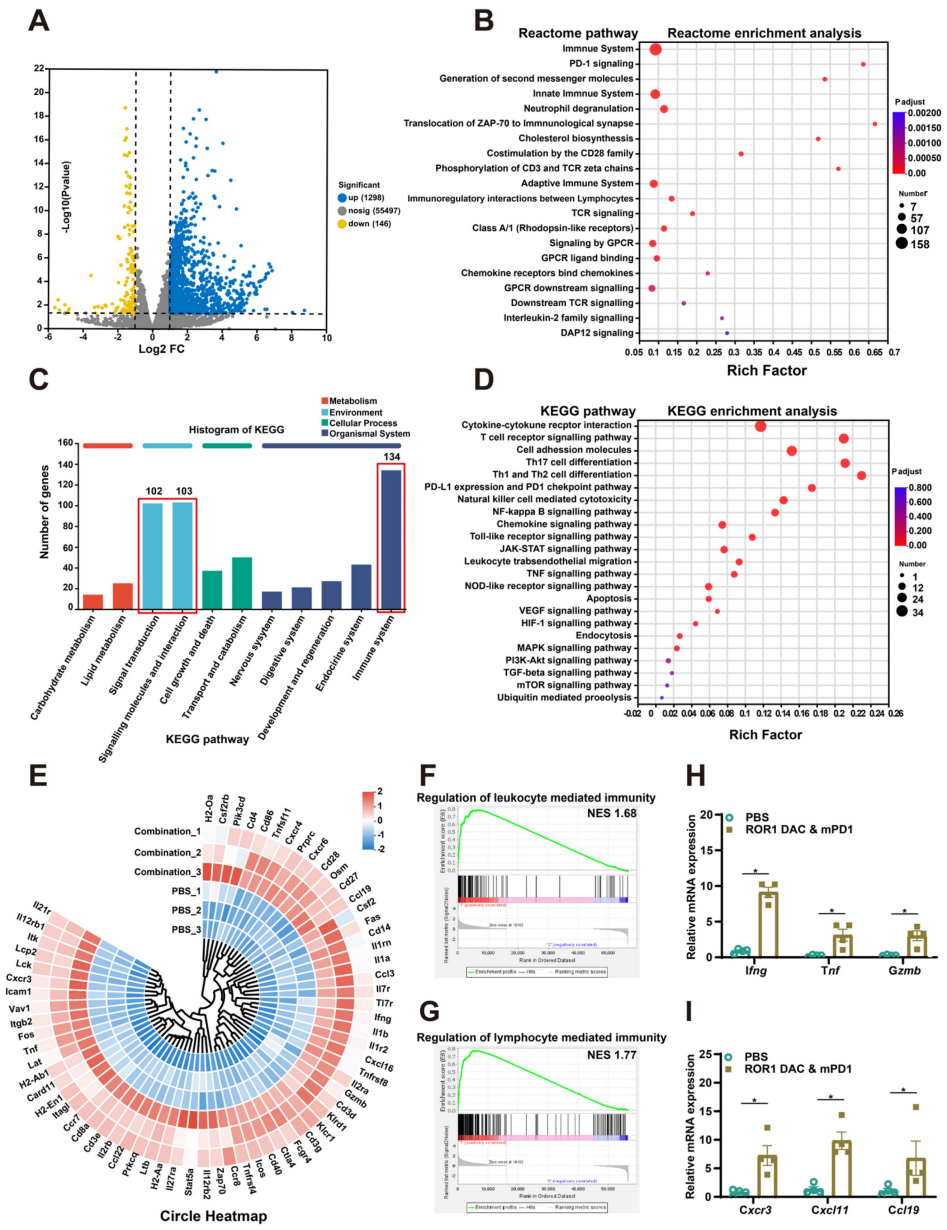
** Transcriptomic analyses of MC38 tumor after ROR1 DAC and αmPD1 combination treatment. (A)** Volcano plot of the differentially expressed genes (DEGs) between the control and combination treatment groups. Yellow and blue spots represent down-and upregulated DEGs (*P* value was 0.05 and log2 (FC)˃2). **(B)** Reactome enrichment analysis of DEGs.** (C)** KEGG annotation analysis of DEGs. The pathways of the immune system, signal transduction, and molecular interactions were annotated. **(D)** KEGG pathway enrichment analysis of DEGs. **(E)** Circle heatmap analysis of the genes implicated in the TME modulation. **(F)** GSEA of regulation of leukocyte mediated immunity gene set. The normalized enrichment score (NES) was 1.68, false discovery rate (FDR) < 0.25 **(G)** GSEA of regulation of the lymphocyte-mediated immunity gene set. NES was 1.77, FDR < 0.25.** (H, I)** Real-time validation for selected genes. Data were analyzed by unpaired t-tests and shown as Mean ± SEM. ns *P* > 0.05; * *P* < 0.05.

To further investigate the therapeutic mechanism of the ROR1 DAC and αmPD1 antibody combination treatment, the total RNA of stripped tumors in PBS and combination groups were extracted and analyzed using RNA sequencing (Table S4). The correlation coefficients for the PBS and combination groups were above 0.925, indicating that the two groups were separated and that the samples within each group were reproducible (Figure S12). A total of 1444 significant DEGs were filtered using the criteria of a *P*value of 0.05 and log2 (FC) ˃2, of which 1298 and 146 genes were upregulated and downregulated, respectively (Figure 6A). Reactome enrichment analysis indicated that these differential genes were associated with immune-related functions (Figure 6B). Analysis of these DEGs through GO enrichment showed that they are related to leukocyte and T cell proliferation, antigen processing and presentation, and cellular extravasation processes (Figure S13). Additionally, KEGG annotation analysis also revealed that the DEGs are associated with the immune system, signal transduction, and interactions (Figure 6C). KEGG enrichment analysis further revealed the detailed antitumor mechanisms of combination treatment by modulating multiple TME-related signaling pathways, including the cytokine and receptor interaction, T cell receptor, NK cell cytotoxicity, and toll-like receptor signaling pathway (Figure 6D). Based on the KEGG enrichment analysis, DEGs were presented in the circle heatmap (Figure 6E). Additionally, GSEA indicated that the combination treatment could positively regulate leukocyte and lymphocyte-mediated immunity, suggesting that strong antitumor immune responses were induced by the combination treatment (Figure 6F, G). To further evaluate immune cell infiltration in both groups, ImmuCellAI_mouse analysis was conducted. The combination treatment group significantly upregulated the infiltration score associated with lower Treg cells and significantly higher CD8-related immune cells and Th1 cells (Figure S14). Compared to the control group, combination treatment significantly increased the expression of I*fng*, T*nf*, G*zmb*, C*xcr3*, C*xcl11* and C*cl19* in the tumor, suggesting a more inflammatory TME (Figure 6H, I). Additionally, treatment with ROR1 DAC alone also affects chemokine gene regulation, such as that of C*xcr3* and C*cl19*, which is consistent with the combination group (Figure S15). Collectively, these results confirm that the combination treatment of ROR1 DAC and αmPD-1 antibody can modulate immune cell-mediated immunity and infiltration, leading to potent antitumor immunity.

### ROR1 DAC exhibits a favorable *in vivo* safety profile

**Figure 7:**
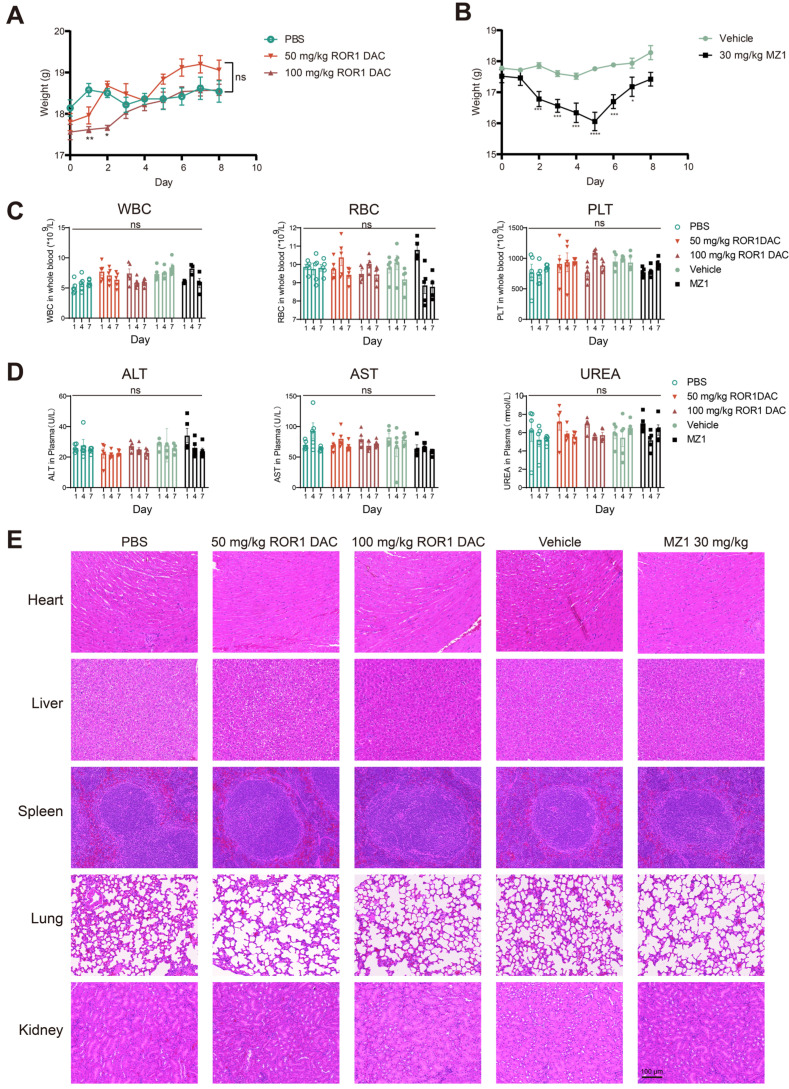
** Safety evaluation of ROR1 DAC on BALB/c mice. (A)** Changes in body weight of ROR1 DAC groups and MZ1 group. **(B)** Mice (n = 5) were intravenously injected with a single dose of PBS, 50 mg/kg ROR1 DAC, 100 mg/kg ROR1 DAC, and intraperitoneally injected with two doses of the vehicle and 30 mg/kg MZ1. Body weight was measured daily. **(C)** Blood routine examination in BALB/c mice. Blood collections were conducted on days 1, 4, and 7. White blood cells (WBC), red blood cells (RBC), and platelets (PLT) were monitored using the Sysmex Hemostasis Analyzer XN-1000V (B1).** (D)** Evaluation of liver and kidney function in BALB/c mice. Blood collections were conducted on days 1, 4, and 7. The plasmas were separated for detection. Alanine aminotransferase (ALT), aspartate aminotransferase (AST), and urea (UREA) were monitored on days 1, 4, and 7 using the Mindray Chemistry Analyzer BS360S.** (E)** H&E staining was performed to evaluate the tissue pathology of major organs. Scale bar: 100 µm. Data were analyzed by One-way ANOVA and shown as Mean ± SEM. ns *P* > 0.05; * *P* < 0.05; **P < 0.01; ***P < 0.001; ****P < 0.0001.

Following the consecutive administration of ROR1 DAC, no significant toxicity was observed in any of the three mouse models. Therefore, we conducted an acute toxicity study to further investigate the safety profile of ROR1 DAC. Weight loss was observed in the 50 mg/kg and 100 mg/kg ROR1 DAC groups during the first two days; however, it recovered by day 3, while more pronounced weight loss occurred in the 30 mg/kg MZ1 group (Figure 7A, B). Certain BET inhibitors can lead to dose-limiting toxicities in clinical trials, including thrombocytopenia, diarrhea, fatigue, and hepatotoxicity 34. In this study, sequential monitoring of routine blood examinations and liver and renal function was conducted. As shown in Figure 7C-D, ROR1 DAC did not significantly alter the levels of white blood cells (WBC), red blood cells (RBC), and platelets (PLT). Furthermore, no significant changes in alanine aminotransferase, aspartate aminotransferase, and urea were observed between the control and ROR1 DAC groups. Notably, RBC counts decreased on days 4 and 7, and fur yellowing (not shown) was observed in the 30 mg/kg MZ1 group, indicating potential toxicity associated with MZ1. Compared to the control group, no obvious lesions were observed in the main organs of the two ROR1 DAC groups (Figure 7E). The results indicate that ROR1 DAC has a better safety profile than the unconjugated MZ1, which could potentially broaden the therapeutic window.

## Discussion

PROTACs are widely used to degrade undruggable targets in cancer therapy, but the poor solubility, permeability, and targeting specificity limit their clinical translational process 35. Considering *in vivo* safety, off-target side effects are also a concern. This study leverages antibodies as PROTAC carriers to overcome these obstacles and enhance antitumor bioactivity.

The stability and efficacy of degrader-antibody conjugates are influenced by the conjugation site, linker steric hindrance, and linker type 36. We compared the effects of different conjugation sites and linker lengths on degradation and *in vitro* efficacy. The conjugates with one mutation site exhibited weaker degradation and cytotoxicity than those with two or three mutation sites, which is consistent with previous reports 11. Previous studies have demonstrated that STEAP1 DACs with non-cleavable linkers provide no significant advantages over DACs with cleavable linkers 37. Given that the non-cleavable linker may enhance stability and reduce off-target toxicity 38, 39, it could serve as an alternative option for conjugation.

Antibody-drug conjugates represent a rational delivery format among aptamer conjugates and peptide conjugates, owing to their antibody-mediated specific targeting ability, internalization, pharmacokinetic properties, and manufacturing flexibility 40. Additionally, the conjugation extended the half-life of PROTAC and enhanced the antitumor efficacy of the molecular equivalent, consistent with the CLL1-degrader conjugate 11. However, unlike the CLL1-degrader conjugate, ROR1 DAC treatment significantly inhibits tumor growth with continuous dosing, potentially due to the modest cytotoxicity and sustained degradation requirements of MZ1. Future investigations could focus on conjugating ROR1 DAC with potent degraders or bispecific antibodies to enhance therapeutic efficacy 41-43.

Several reasons underscore the advantage of ROR1-targeting conjugates. ROR1 is notably absent in normal tissues following embryonic development, yet it is expressed in a range of hematological and solid tumors, suggesting a broad range of potential therapeutic applications. Additionally, ROR1 is likely to have superior endocytic activity in comparison with HER2, potentially enhancing the efficacy of ROR1-targeting conjugates in the delivery of PROTAC to cancer cells. Notably, trastuzumab, a prevalent HER2-targeting antibody, has been found to either poorly facilitate HER2 endocytosis or to be rapidly returned to the cell membrane post-endocytosis. Furthermore, the corresponding expression of ROR1 and BRD4 targets on the same cells is essential for the development of DACs. Lastly, ROR1 signaling has been implicated in cell survival and migration in cancer cells. ROR1 DAC may not only inhibit these processes but also has the potential to modulate the tumor microenvironment, which can be particularly beneficial when combined with immunotherapies like PD-1 blockade 44. In addition to synergistic effects with immune checkpoint inhibitors, degradation of BRD4 may enhance the antitumor effects of chemotherapy, positioning ROR1 DAC as a promising addition to cancer treatment regimens 45.

BET proteins have been reported to participate in the transcriptional regulation of pro-inflammatory and immunomodulatory genes, influencing the remodeling of the tumor microenvironment 46. Therefore, we propose ROR1 DAC as a combination therapy with immune checkpoint inhibitors. The combination of αmPD-1 mAb with ROR1 DAC enhanced the suppression of tumor growth compared to ROR1 DAC monotherapy, consistent with studies of JQ1 and PD-1 mAb combinations in non-small cell lung cancer 47. Li *et al.* reported that BRD4 inversely correlates with the infiltration of CD8^+^ T cells in esophageal cancer 48. We observed an increase in intra-tumoral T cell infiltration and splenic CD8^+^ T cells induced by ROR1 DAC, consistent with the idea that epigenetic changes can promote the infiltration and re-population of immune cells 49. Suppression of PD-L1 by BET inhibitors has been reported in lymphomas and ovarian cancer 33, 50. Our results demonstrated that the combination treatment significantly upregulated mRNA levels of I*fng*, T*nf,* and C*xcl11,* which are pro-inflammatory biased mediators. Given that BET proteins are also involved in immune surveillance and the secretion of cytokines and chemokines, exploring the application of ROR1 DAC for potential immunological benefits in other therapies warrants further investigation 51-53.

The biodistribution of ADC drug primarily depends on its antibody component, which generally results in fewer side effects than unconjugated drugs. We also demonstrated that the safety profile of MZ1 improved after conjugation. Compared to MZ1, ROR1 DAC did not cause severe dosing toxicity in three mouse models or induce acute toxicity after high-dose administration. Additionally, changes in dosage formulations may further enhance the safety profile. Previous reports suggest that sustained BRD4 suppression could impair immune surveillance, hematopoiesis, and memory consolidation 54-56. Thus, although ROR1 targeting may alleviate off-tumor toxicity, long-term administration of the degrader should be avoided.

## Conclusions

In summary, we developed a ROR1-targeting degrader-antibody conjugate and characterized its specificity, BRD4 degradation, internalization, and cytotoxicity *in vitro*. Furthermore, due to its long half-life and tumor-targeting properties, ROR1 DAC exhibited superior *in vivo* antitumor activity and a safety profile. Given that BRD4 plays a crucial role as an immune gene regulator, we assessed the antitumor efficacy of ROR1 DAC when combined with αmPD-1 mAb. The combination treatment effectively inhibited tumor progression and elicited the immune response. Overall, our research provides practical insights into the development of degrader-antibody conjugates for cancer treatment.

## Supplementary Material

Supplementary figures and tables.

## Abbreviations

ADC: antibody-drug conjugate
ALT: alanine aminotransferase
AST: aspartate aminotransferase
APC: allophycocyanin
αmPD1: anti-mouse programmed cell death protein 1
BCA: bicinchoninic acid assay
BET: bromodomain and extra-terminal
BRD4: bromodomain-containing protein 4
CCK-8: cell counting kit-8
DAC: degrader-antibody conjugate
DEGs: differentially expressed genes
DHAA: dehydroascorbic acid
ELISA: enzyme-linked immunosorbent assay
FBS: fetal bovine serum
FDR: false discovery rate
FITC: fluorescein isothiocyanate
GO: gene ontology
GSEA: gene set enrichment analysis
H&E staining: hematoxylin and eosin staining
KEGG: kyoto encyclopedia of genes and genomes
MFI: median fluorescence intensities
NES: normalized enrichment score
PBMCs: peripheral blood mononuclear cells
PD-L1: programmed cell death ligand-1
PE: phycoerythrin
PI: propidium iodide
PK: pharmacokinetic
PLT: platelet
POI: protein of interest
PROTAC: proteolysis targeting chimera
PVDF: polyvinylidene fluoride
RBC: red blood cell
RNA-seq: rna-sequencing
ROR1: receptor tyrosine kinase-like orphan receptor 1
SDS-PAGE: sodium dodecyl sulfate-polyacrylamide gel electrophoresis
TECP.HCl: tris (2-carboxyethyl) phosphine hydrochloride
THIOMABs: antibodies with engineered reactive cysteine residues
TPD: targeted protein degradation
UREA: urea
VHL: von hippel-lindau
WBC: white blood cell
