# Supplementary material for: A novel ROR1-targeting antibody-PROTAC conjugate promotes BRD4 degradation for solid tumor treatment
Source: Theranostics. 2025 Jan 1;15(4):1238–54. doi: 10.7150/thno.102531 (PMC11729552; doi:10.7150/thno.102531)
Supplement: Supplementary file 1 — Supplementary figures and tables. [file thnov15p1238s1.pdf]

Supplementary materials

Original article

A novel ROR1-targeting antibody-PROTAC conjugate promotes BRD4 degradation for solid tumor treatment

Lei Wang[a], Yong Ke[a], Qunye He[a], Pameila Paerhati[a], Weiliang Zhuang[a], Yali Yue[a], Junjun Liu[a], Jiawei Zhang[a], Lulu Huang[a], Qiang Yin[a], Huifang Zong[b], Jianwei Zhu[a,b,*], Baohong Zhang[a,*]

[a]*Engineering Research Center of Cell & Therapeutic Antibody, Ministry of Education, School of Pharmacy, Shanghai Jiao Tong University, Shanghai 200240, China*

[b]*Jecho Institute, Co. Ltd, Shanghai 200240, China*

*Corresponding authors.

E-mail addresses: bhzhang@sjtu.edu.cn (Baohong Zhang), jianweiz@sjtu.edu.cn (Jianwei Zhu).

Materials and Methods

Supporting figures (Figure S1–S15)

Supporting tables (Table S1–S4)

Methods

Serum stability assay of ROR1 DAC

The ROR1 DAC was diluted in 1 mL mouse serum with a concentration of 200 μg/mL and incubated at 37 °C under 5% CO_2. Samples were taken on days 0, 1, 3, 7, and 14 and frozen at -80 °C. 25 μL of each sample were taken, digested with 1 μL Endo S (20 U/μL, NEB, Beverly, MA, USA) at 37 °C for 1 h, captured with 20 μL Biotin-SP-conjugated AffiniPure Donkey anti-human (H+L) antibody (Jackson Lab, West Grove, PA, USA) at 37 °C for 1 h and room temperature for 1 h, incubated with 60 μL streptavidin magnetic beads (MCE, Monmouth Junction, NJ, USA) at room temperature for 1.5 h, washed PBST (0.05%), PBS and ddwater for two times, eluted with 55 μL formic acid in water, reduced with TCEP (final concentration 200 mM) at 37°C for 0.5 h and centrifuged before LC/MS detection. Samples were detected using ACQUITY UPLC Protein BEH C4 Column with 0.1% formic acid in water as mobile phase A and 0.1% formic acid in acetonitrile as mobile phase B, followed by Xevo G2 Q-Tof mass spectrometer (Waters, Manchester, UK) coupled with nanoAcquity UPLC (Waters, Manchester, UK). Data were analyzed using Biopharmalynx software (Waters, Manchester, UK). DAR value was calculated by the equation: Average DAR = LC1/(LC0+LC1) × 2 + HC1/(HC0+HC1+HC2) × 2 + HC2/(HC0+HC1+HC2) × 4.

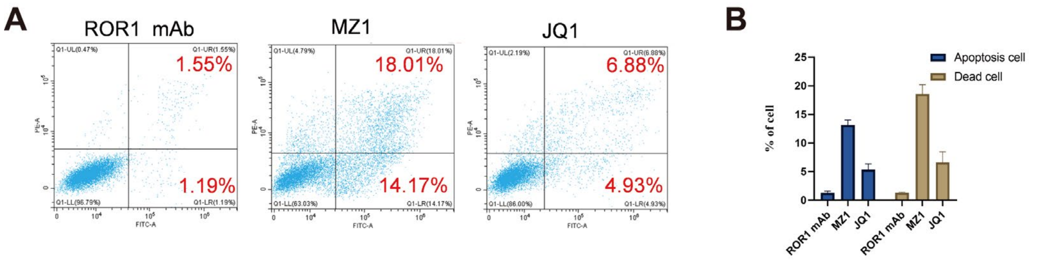

Figure S1 Apoptosis activity of MZ1 and JQ1 on PC3 cells. (A) Apoptosis assay of PC3 cells by flow cytometry. Cells were incubated with 100 nM ROR1 mAb, 1 μM MZ1, and for four days. Cells were collected and subjected to analysis of Annexin V-PI. The histogram of dead cells and apoptosis cells were counted using GraphPad Prism 8.3.0. **(B)** The histogram of early and late apoptotic cells.

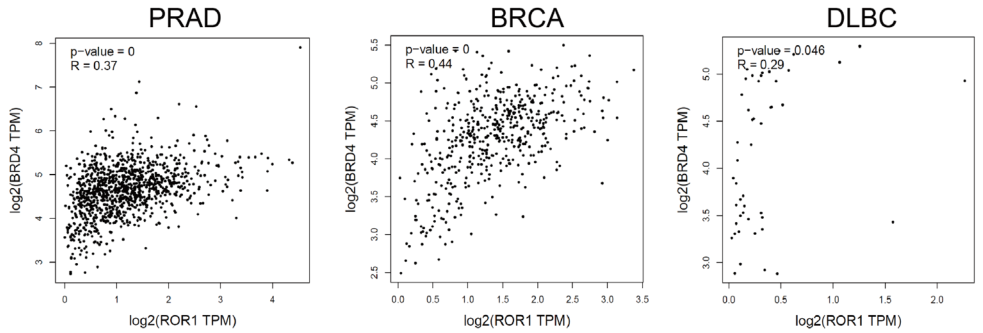

Figure S2 Correlations between BRD4 and ROR1 expression level across TCGA (The Cancer Genome Atlas cancers) on GEPIA2 (Gene Expression Profiling Interactive Analysis, version 2) website online analysis. PRAD: Prostate adenocarcinoma, BRCA: Breast invasive carcinoma, DLBC: Lymphoid Neoplasm Diffuse Large B-cell Lymphoma. log2 [TPM(Transcripts per million)+1] for log-scale, $P < 0.05$

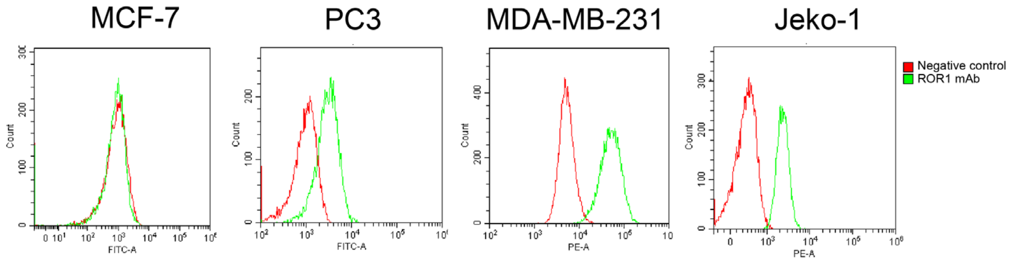

Figure S3 ROR1 antigen expression level on different tumor cell lines by flow cytometry.

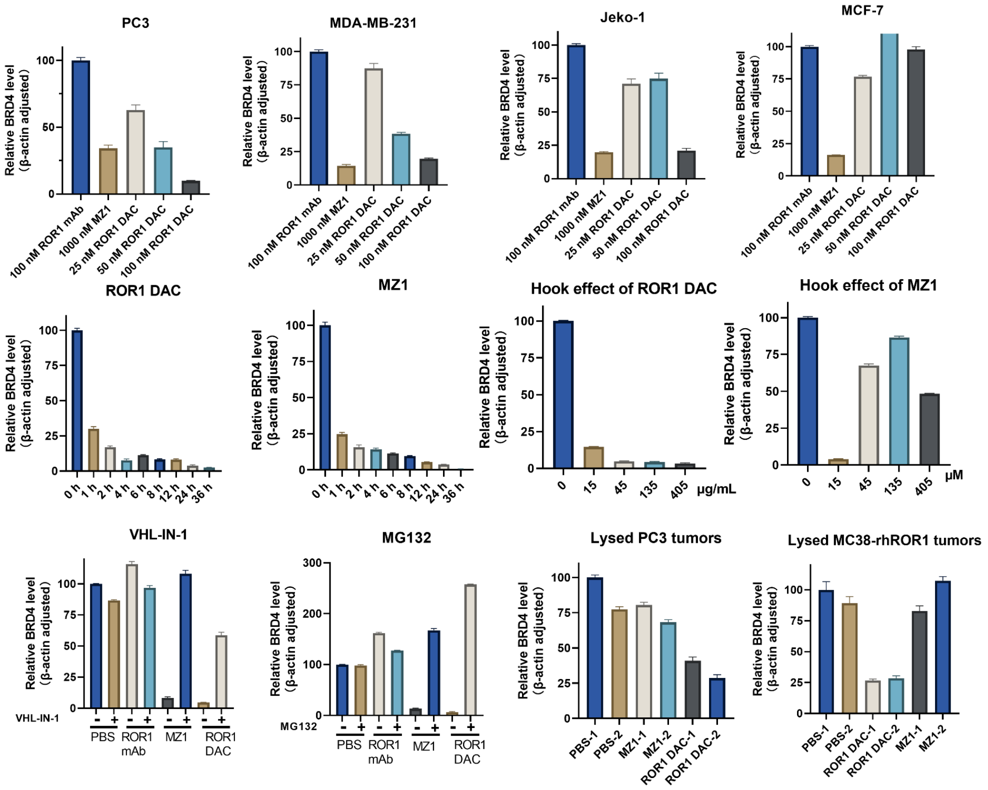

Figure S4 Quantification of western blot data. The grayscale intensities of the western blot images were determined using ImageJ software, and the resulting data were plotted using GraphPad Prism version 8.3.0 software.

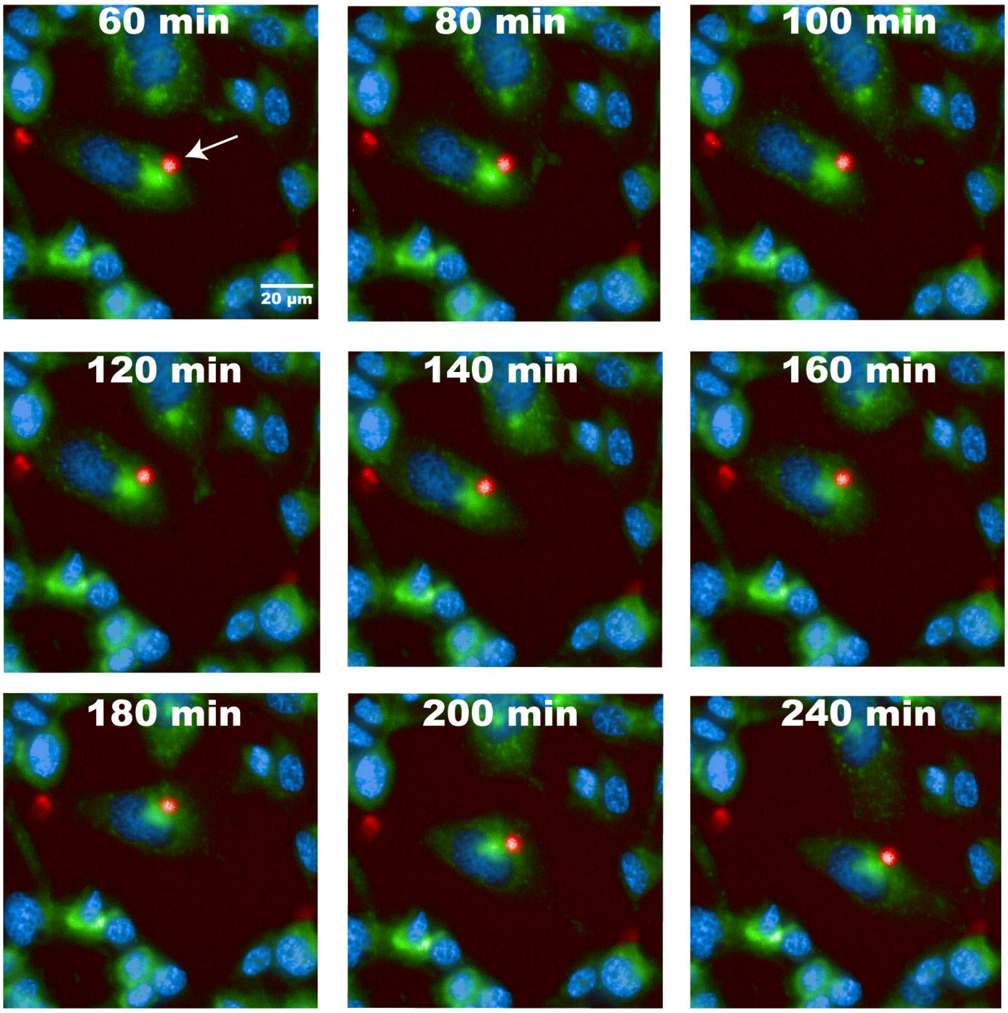

Figure S5 Temporal analysis of ROR1 DAC internalization. PC3 cells were stained with LysoSensor for 40 min and Hoechst solution for 10 min, followed by washing with pre-warmed PBS. Subsequently, incubated with 15 nM ROR1 DAC and imaged at the indicated time. Scale bar: 20 μm. Images of live cells were captured by Operetta CLS.

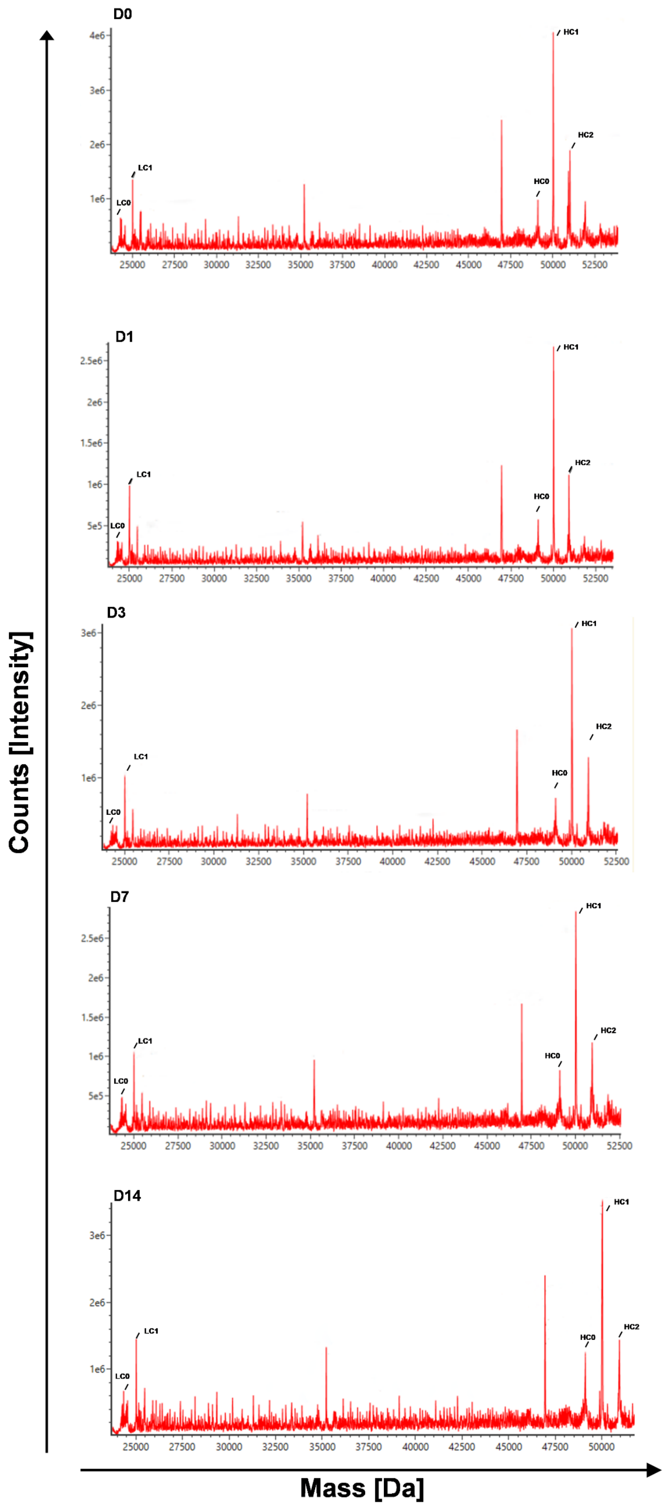

Figure S6 LC/MS analysis of ROR1 DAC serum stability. The ROR1 DAC was incubated in mouse serum at time points of 0, 1, 3, 7, and 14 days. Subsequently, the conjugates were captured using Biotin-SP-conjugated AffiniPure Donkey anti-human (H+L) antibody and streptavidin magnetic beads. The stability of the ROR1 DACs in serum was assessed by liquid chromatography-mass spectrometry (LC/MS) analysis.

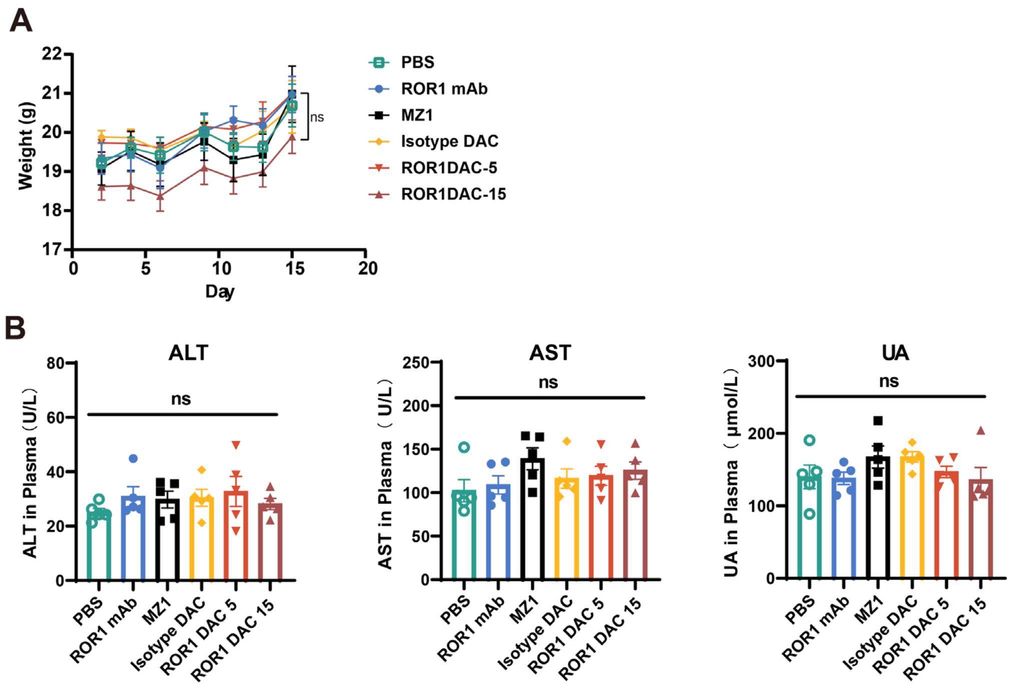

Figure S7 Monitoring of body weight and liver and kidney functions on PC3 xenograft mouse model. (A) Body-weight changes of different groups. **(B)** Detection of liver and kidney functions of different groups on PC3 xenograft mouse model. Plasmas were collected on the final day of the experiment. ALT, AST, and UREA were detected using Mindray Chemistry Analyzer BS360S.

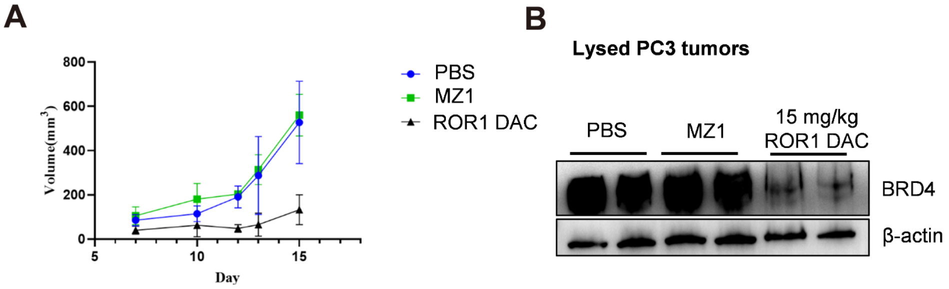

Figure S8 The western blotting analysis of the ROR1 DAC in tumors. (A) PC3 tumor volume changes in different groups. 5×10^6 cells were implanted on the right flank of the mouse. When the tumor volume reached 50-100 mm^3, mice were treated with PBS and ROR1 DAC (15 mg/kg) every five days and MZ1 (15 mg/kg) every two days. **(B)** BRD4 level detection on lysed PC3 tumors.

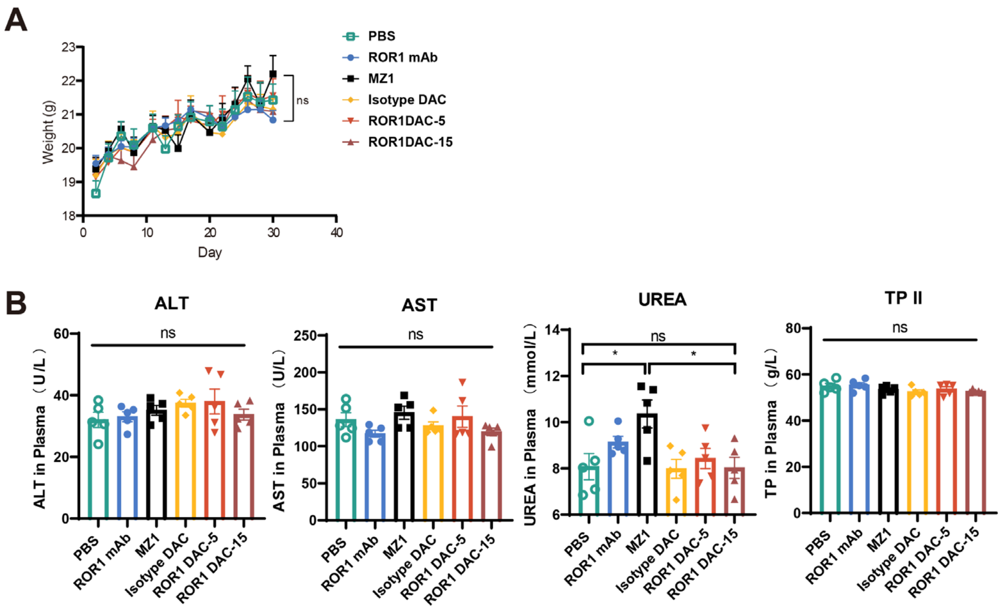

Figure S9 Monitoring of body weight and liver and kidney functions on MDA-MB-231 xenograft mouse model. (A) Body-weight changes of different groups. **(B)** Detection of liver and kidney functions of different groups on MDA-MB-231 xenograft mouse model. Plasmas were collected on the final day of the experiment. ALT, AST, UREA, and TPⅡ were detected using Mindray Chemistry Analyzer BS360S.

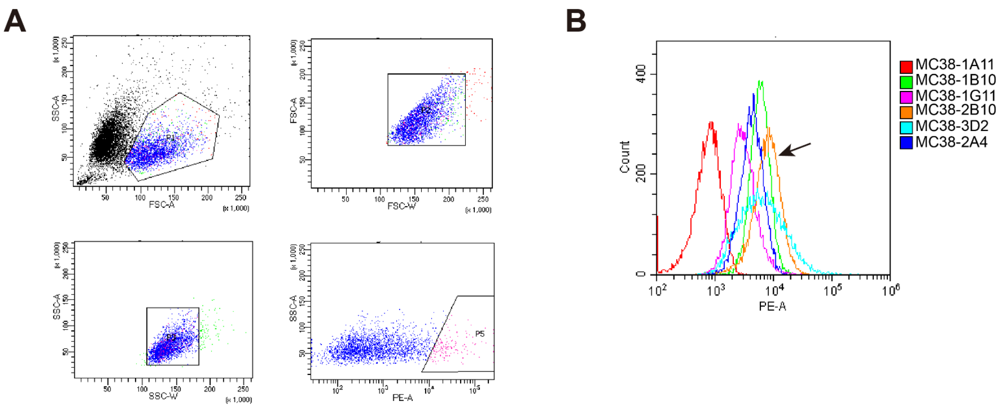

Figure S10 The gate to identify MC38-rhROR1 positive cell. (A) The top 5% of the highest fluorescence intensity were gated for single-cell sorting. **(B)** The 2B10, with the second-highest fluorescence intensity, was selected for subsequent experiments.

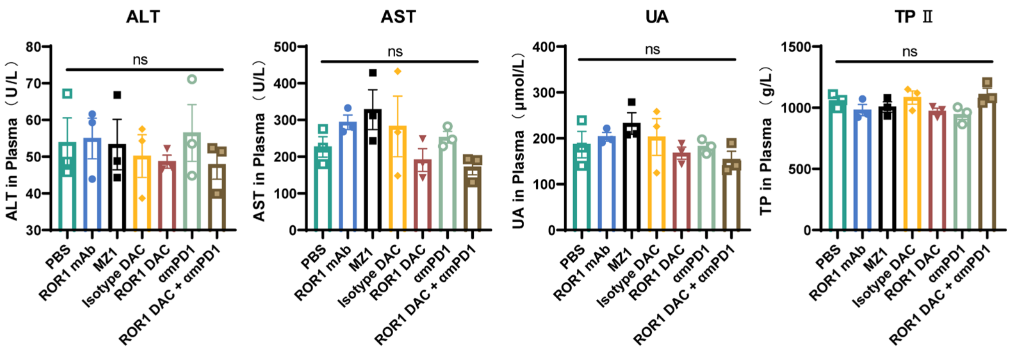

Figure S11 Detection of liver and kidney functions of different groups on MC38-rhROR1 xenograft mouse model. Plasmas were collected Plasmas were collected on the final day of the experiment. ALT, AST, UA, and TPⅡ were detected using Mindray Chemistry Analyzer BS360S.

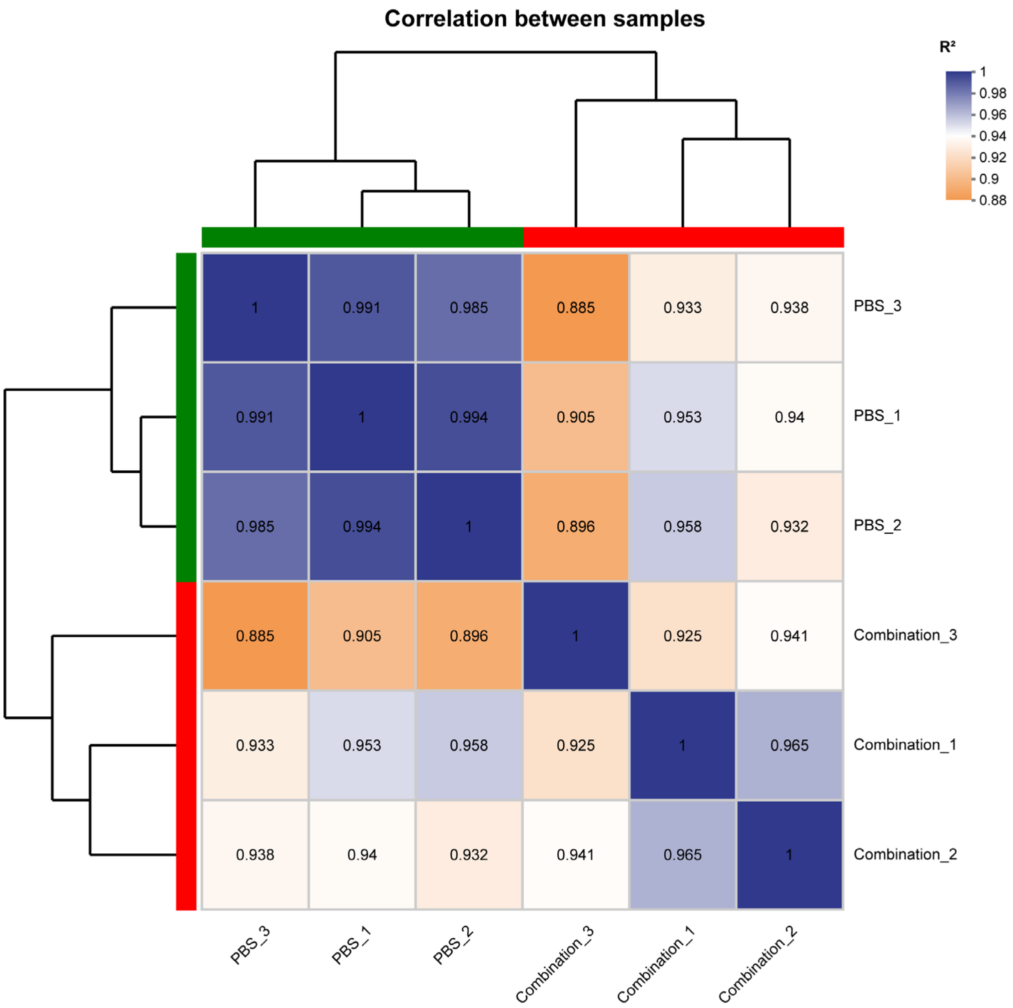

Figure S12 The heatmap diagram of correlation analysis. Coefficient values among three biological replicates of control and combination treatment groups were shown. Maximum correlation is represented by the dark blue color, with the minimum correlation indicated by the orange color.

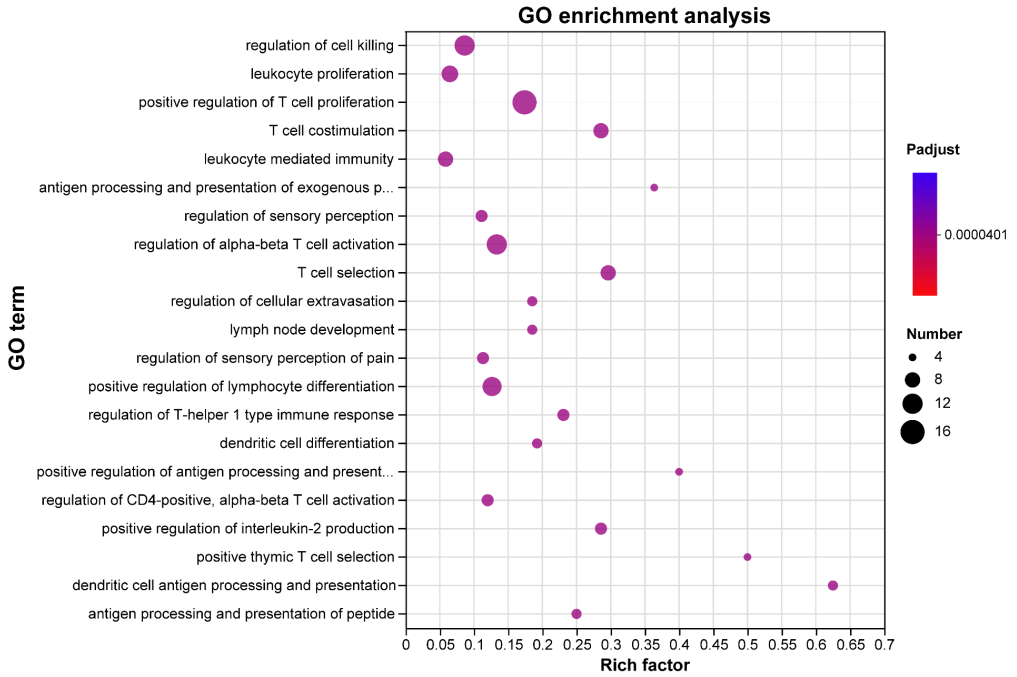

Figure S13 GO pathway enrichment analysis of DEGs.

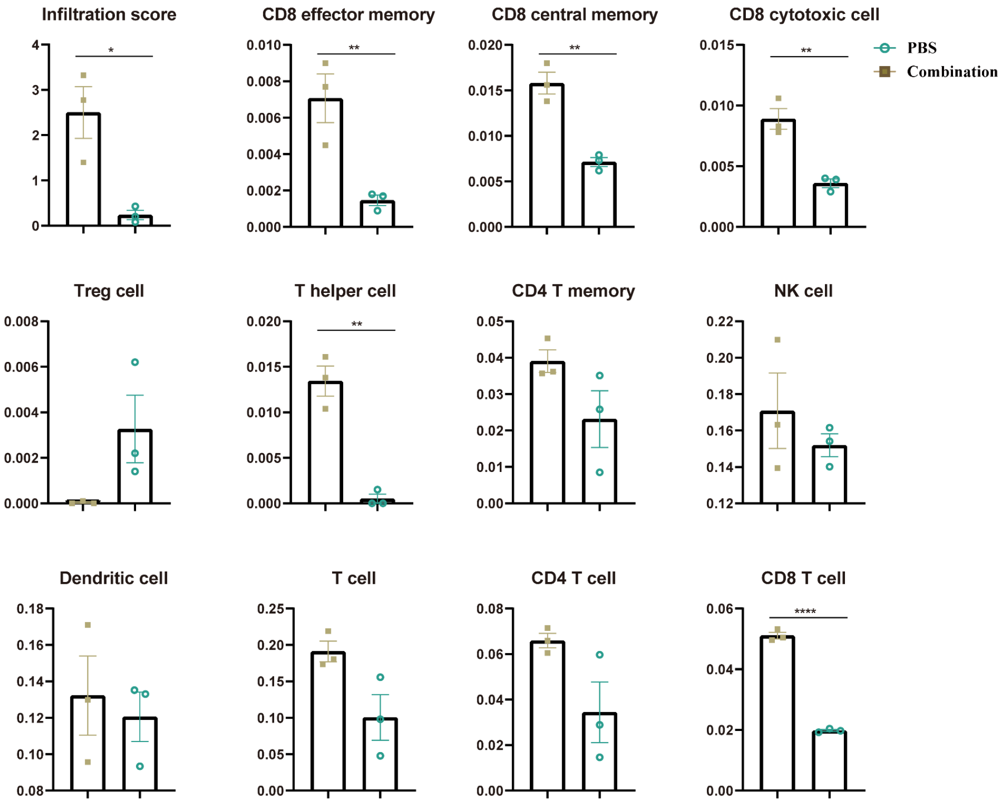

Figure S14 Immune cell infiltration analysis using ImmuCellAI_mouse. Infiltration score and abundance of the eleven major immune cell types between control and combination treatment groups were analyzed.

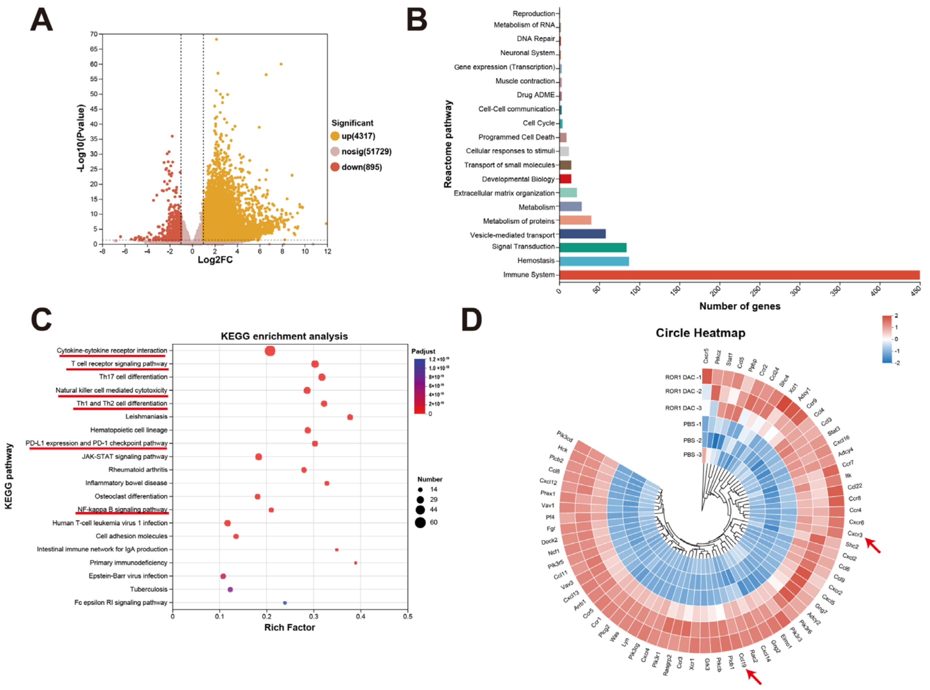

Figure S15 Transcriptomic analysis of MC38 tumor after ROR1 DAC and PBS treatment. (A) Volcano plot of the differentially expressed genes (DEGs) between the control and ROR1 DAC treatment groups. Yellow and blue spots represent down-and upregulated DEGs (Pvalue was 0.05 and log2 (FC) >2). **(B)** Reactome enrichment analysis of DEGs. **(C)** KEGG pathway enrichment analysis of DEGs. **(D)** Circle heatmap analysis of the genes implicated in the TME modulation.

Table S1 Primer sequences for quantitative real-time PCR analysis.

Gene	Forward	Reverse
I*nfg*	ATGAACGCTACACACTGCATC	CCATCCTTTTGCCAGTTCCTC
C*xcr3*	TACGATCAGCGCCTCAATGCCA	AGCAGGAAACCAGCCACTAGCT
T*nf*	CCCTCACACTCAGATCATCTTCT	GCTACGACGTGGGCTACAG
C*xcl1*	CCGAGTAACGGCTGCGACAAAG	CCTGCATTATGAGGCGAGCTTG
G*zmb*	CAGGAGAAGACCCAGCAAGTCA	CTCACAGCTCTAGTCCTCTTGG
C*cl19*	TCGTGAAAGCCTTCCGCTACCT	CAGTCTTCGGATGATGCGATCC
G*apdh*	TCTCCTGCGACTTCAACA	TGGTCCAGGGTTTCTTACT

Table S2 Degradation and cytotoxicity summary of ROR1 conjugates with different mutation sites.

Mutations	Light chain	Heavy chain	DC_{50} (nM)	IC_{50} (nM)
K149C	K149C	-	237.8	128.0
LY	-	L174C, Y373C	46.58	76.71
KV	-	K274C, V422C	54.28	74.64
KLY	K149C	L174C, Y373C	26.22	57.86

-no mutations.

Table S3 IC_{50} of ROR1 DAC in different cell lines.

IC_{50} (nM)	PC3	MDA-MB-231	Jeko-1	MCF-7
ROR1 mAb	ND	ND	ND	ND
MZ1	108.7	82.09	ND	32.92
ROR1 DAC	102.7	93.35	86.81	ND

ND, no significant cytotoxicity was observed.

Table S4 Quality tests of total RNA.

Sample	Concentration (ng/μL)	Quality (μg)	OD260/OD280	OD280/OD260
PBS_1	1913.16	66.96	1.96	2.25
PBS_2	753.65	64.06	1.93	2.21
PBS_3	1936.18	67.77	1.97	2.21
Combination_1	806.26	28.22	1.91	2.26
Combination_2	692.82	24.25	1.90	2.25
Combination_3	1394.85	48.82	1.95	2.22
